# Demographic and geographical determinants of human olfactory perception of 909 individuals inhabiting 16 regions

**DOI:** 10.1016/j.isci.2025.113455

**Published:** 2025-09-18

**Authors:** Eva Drnovsek, Nixon M. Abraham, Jancy N. Abraham, Rafieh Alizadeh, Ines Aloulou, Lixin Chen, Ma. Lourdes Berioso Enecilla, Marco Aurélio Fornazieri, Johannes Frasnelli, Juan Martin Fuselli, Fatima Gansatao, Cagdas Guducu, Anna Kristina Hernandez, Marlise K. Hofer, Salina Husain, Reda Kamel, Vitoria F. Khouri, Francesco Loy, Mehmet K. Mahmut, Daniel Marek, Carla Masala, Natália Medeiros Dias Lopes, Élizabeth Michaluk, Imen Miri, Marjan Mirsalehi, Plamena Miteva, Anasuha Musa, Hanène Naija, Keigo Nakaachi, Michal Pieniak, Jayant M. Pinto, Patricia Portillo Mazal, Ahmed Radwan, Farhad Rafiei, Devesh Rawat, Aleksandra Reichert, Henrique O. Scussiatto, Hozifa Alsaid Sheta, Sharanya M. Thodupunoori, Brianna J. Turner, Hangying Wu, Fiona Wylie, Ayaho Yoshino, Laiquan Zou, Barbara Zyzelewicz, Gregory N. Bratman, Asifa Majid, Thomas Hummel, Anna Oleszkiewicz

**Affiliations:** 1 Smell & Taste Clinic, Department of Otorhinolaryngology, Faculty of Medicine Carl Gustav Carus, Technische Universität Dresden, Dresden, Germany; 2 Laboratory of Neural Circuits and Behaviour (LNCB), Department of Biology, Indian Institute of Science Education and Research (IISER), Pune, Maharashtra, India; 3 Department of Life Sciences, Centre of Excellence in Epigenetics, Shiv Nadar Institution of Eminence, Gautam Buddha Nagar, Uttar Pradesh, India; 4 ENT and Head and Neck Research Center and Department, The Five Senses Health Institute, School of Medicine, Iran University of Medical Sciences, Tehran, Iran; 5 PMR Department A, Institut Kassab, Manouba, Tunisia; 6 Chemical Senses and Mental Health Laboratory, Department of Psychology, School of Public Health, Southern Medical University, Guangzhou, Guangdong, China; 7 St. Luke’s Medical Center, Quezon City, Manila, Philippines; 8 Universidade Estadual de Londrina (UEL), Londrina, Brazil; 9 Pontifícia Universidade Católica do Paraná (PUC-PR), Curitiba, Brazil; 10 Department of Anatomy, Université du Québec à Trois-Rivières, Trois-Rivières, QC, Canada; 11 Hospital Naval Dr. Pedro Mallo, Buenos Aires, Argentina; 12 East Avenue Medical Center, Quezon City, Manila, Philippines; 13 Dokuz Eylul University, Faculty of Medicine, Department of Biophysics, Izmir, Türkiye; 14 Department of Otolaryngology - Head and Neck Surgery, Philippine General Hospital, University of the Philippines - Manila, Manila, Philippines; 15 Department of Psychology, University of Victoria, Victoria, BC, Canada; 16 Department of Otorhinolaryngology – Head and Neck Surgery, Faculty of Medicine, Universiti Kebangsaan Malaysia, Kuala Lumpur, Malaysia; 17 Department of Otolaryngology, Cairo University, Cairo, Egypt; 18 Department of Biomedical Sciences, University of Cagliari, SP 8 Cittadella Universitaria, Monserrato, Italy; 19 Food, Flavour and Fragrance Lab, School of Psychological Sciences, Macquarie University, Sydney, NSW, Australia; 20 Institute of Psychology, University of Wroclaw, Wroclaw, Poland; 21 GEM - Centro De Excelencia Em Pesquisa, Ensino E Atencao A Saude, Londrina, Brazil; 22 Laboratory of Clinical Psychology, Intersubjectivity and Culture, Faculty of Medicine in Tunis, Tunis, Tunisia; 23 Department of Otolaryngology, Nippon Medical School, Tokyo, Japan; 24 Department of Surgery, Pritzker School of Medicine, the University of Chicago Biological Sciences Division, Chicago, IL, USA; 25 Hospital Italiano de Buenos Aires, Buenos Aires, Argentina; 26 School of Environmental and Forest Sciences, University of Washington, Seattle, Washington, USA; 27 Department of Experimental Psychology, University of Oxford, Oxford, UK

**Keywords:** Natural sciences, Biological sciences, Neuroscience, Clinical neuroscience, Sensory neuroscience

## Abstract

The hedonic perception of odors is similar worldwide. However, our perception of smells is much more than just determining whether an odor is pleasant or not. Here, we expanded this assessment by recruiting 909 people from 16 regions of the world and measuring 12 perceptual dimensions (e.g., pleasantness, intensity, edibility), which were aggregated into an olfactory perceptual fingerprint. We used two fingerprints: descriptor-specific and odor-specific. Age, gender, and region explained 1.1%, 0.3%, and 9.6% of variance in the descriptor-specific fingerprints, respectively. Similarly, age, gender, and region explained 0.5%, 0.3%, and 8.2% of variance in the odor-specific fingerprints. Interestingly, odor intensity was more regionally dependent than pleasantness. Thus, olfactory perception across the globe may be better differentiated by odor intensity than pleasantness. Although there is some influence of individual and cultural backgrounds, human perception of odors appears to be quite similar worldwide, even when assessed using 12 perceptual dimensions.

## Highlights

• Olfactory perception of 909 people from 16 regions was evaluated
• Human perception of odors appears to be quite similar worldwide
• Olfactory perception worldwide is better differentiated by intensity than hedonics
• Age and gender had a limited impact on individual perception of odors

## Graphical abstract

**Figure:**
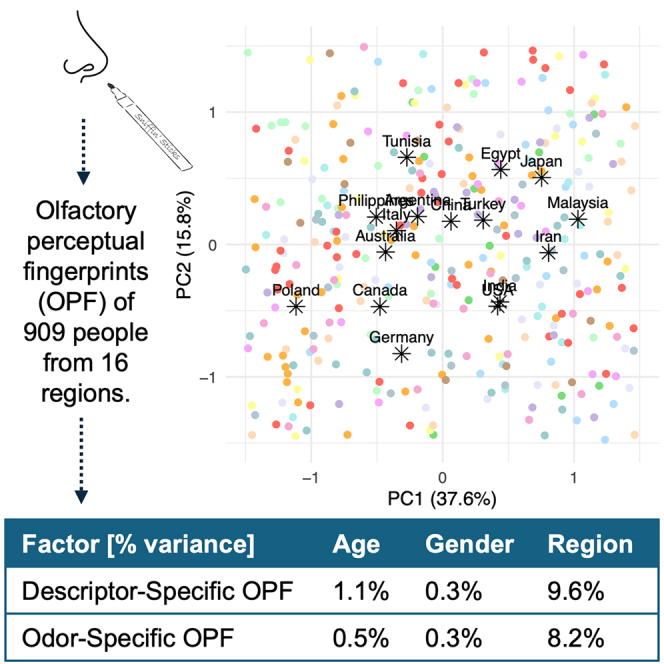

## Introduction

Olfactory perception varies between individuals, and the sources of this variance are not coherently cataloged. It is a matter of scientific debate whether the perception of odors is an objective or subjective cognitive process. Some studies suggest that the ways we perceive odors are universal and can be predicted from the molecular structure of the odorants.^1^^,^^2^^,^^3^ Yet, it has been shown that the perception of odors changes with age, gender,^4^ culture,^5^ physiological state,^6^ and contextual variables such as odor labels,^7^ cross-modal interaction,^8^ or repeated exposure.^9^ It appears that olfactory perception is a complex and multi-faceted phenomenon. How we perceive odors may be a product of the nature of an odor, but also demographic characteristics, health status, and individual experience with odors. Large-scale studies on olfactory perception are needed to empirically confirm this assumption. Psychophysiological research across cultures, among demographically and health-diverse groups, is therefore essential to better understand the determinants of our perception of odors. Some efforts have already been made in this direction, but the perspective on human odor perception is still far from complete.

Thus far, cross-cultural research on olfactory perception focused mainly on odor pleasantness, which is considered to be the principal characteristic driving odor perception and behavioral responses to olfactory stimuli.^3^^,^^10^^,^^11^^,^^12^ It was assumed that it should vary across the globe due to differential chemosensory experiences, which emerge in contact with various odors, including food-related odors, flora specific to a given region, culturally diverse hygienic rituals and cosmetics, exposure to airborne pollutants, individual health, and so on. Contrary to these expectations, culture explained only 6%–7% of the variance of odor pleasantness ratings among hunter-gatherers and horticulturalists.^13^^,^^14^ Similar results were found in children aged 5–8 years from 18 countries of the Western, Educated, Industrialized, Rich, and Democratic world.^15^

However, olfactory perception is much more than just determining whether an odor is pleasant or not.^16^ It also includes other perceptual dimensions such as odor intensity, edibility, and familiarity. The National Geographic Smell Survey in 1986 included 1.2 million people from ∼80 countries aggregated in nine regions who rated odor pleasantness and intensity of six odors. They found odor-specific differences in both odor pleasantness and intensity between these regions.^5^ Furthermore, in a study comparing Japanese and German women, odor-specific differences were observed in odor pleasantness, intensity, familiarity, and edibility.^17^ In another study, people from Singapore rated odors as less familiar, less intense, and less pleasant than people from Geneva and Liverpool, although both “commonly familiar odors” and odors specific to Singapore were included.^18^ To more comprehensively map perceptual determinants of odors, it is necessary to use a broader set of descriptors. To this end, an olfactory perceptual fingerprint (OPF) has been proposed.^19^

To capture one’s OPF, an individual is asked to rate a set of odors using several perceptual descriptors or dimensions (such as odor pleasantness, intensity, edibility). OPF is a psychophysiological measure of individual variability in olfactory perception. Because the OPF reflects our genetic^20^ and health profiles,^21^^,^^22^ it is believed to have the potential to identify individuals. Thus, it has been named a “fingerprint.”^20^ OPF entails psychophysical assessments of multiple odors along multiple dimensions using a visual analogue scale. Thus, OPF is a relatively comprehensive index of how the world smells to an individual. Although relatively understudied, already two ways of calculation have been proposed.

In 2015, Secundo et al. proposed an odor-specific, descriptor-independent OPF. This is a vector with *N* × (*N* − 1)/2 (where *N* is the number of odors) components of pairwise odor similarities, thus making it odor-specific. An advantage of this method is that it does not rely on interindividual agreement on the meaning of the perceptual descriptors (such as aromaticity) and is therefore descriptor-independent. Importantly, Secundo et al. linked this OPF to one’s genetic information, suggesting that our individual perception of odors is unique. This OPF is explained in detail in STAR Methods (see also Figure S1) and is hereafter referred to as odor-specific OPF.

**Figure 1:**
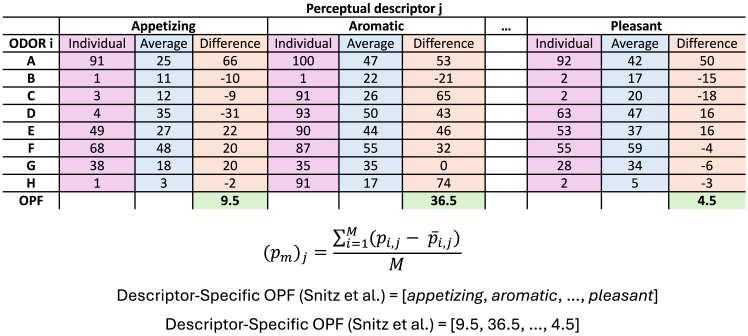
The descriptor-specific OPF for one participant Perceptual ratings by one individual (pink) and average ratings among all participants (blue) for each perceptual descriptor per odor are shown. Additionally, the differences between individual and average ratings are calculated (orange). At the end, the differences are averaged across all odors for each perceptual descriptor, which represents one OPF descriptor (green). Equation for one OPF component (or perceptual descriptor j): participant m rates M odorants. $p_{i,j}$ is the participant’s rating for odorant *i* along a descriptor *j*. ${\bar{p}}_{i,j}$ is the average rating for odorant *i* along a descriptor *j* among all participants.

Next, in 2022, Snitz et al. proposed another, descriptor-specific, odor-independent OPF. This is an *N*-dimensional vector (where *N* is the number of perceptual descriptors). It has been shown to be odor-independent, meaning that it allows comparison of olfactory perception among people who rated different odors using the same perceptual descriptors.^22^ It is described in detail in STAR Methods (see also Figure 1), and hereafter, we refer to this OPF as descriptor-specific OPF.^19^

To date, the studies have focused on the descriptor-specific OPFs of patients with olfactory impairments. However, these two approaches have the potential to study the perception of odors among the general population in a more comprehensive and systematic manner than it has been studied thus far. To begin with, it is unknown to what extent the OPFs vary among healthy people inhabiting different regions of the world or having various experiences with odors. The OPF appears to be a good tool to shed light on the poorly understood phenomenon of cultural and demographical variability in olfactory perception.

This explorative cross-regional study aimed to examine which region and demographics determine the OPF. As our goal was to compare multiple perceptual dimensions across several odors in different world regions, we decided to primarily follow the descriptor-specific OPF concept. However, exploratory analysis using the odor-specific OPF is also reported in the Supplemental Information. We did not have any hypotheses regarding pairwise comparisons of the regions. Regions for this study are meant to represent a sample of culturally diverse populations and have been selected on the basis of convenience, with the prerequisite of a local research team being experienced in chemosensory testing. With this sampling strategy, we hoped to quantify the global variability in the OPFs rather than draw conclusions about differences between cultures in the perception of odors. To this end, we conducted a multi-center study on the OPF in men and women of various age groups inhabiting 18 locations across six continents.

## Results

### Region-related differences explained 10% of the variance in the descriptor-specific OPFs

**Figure 2:**
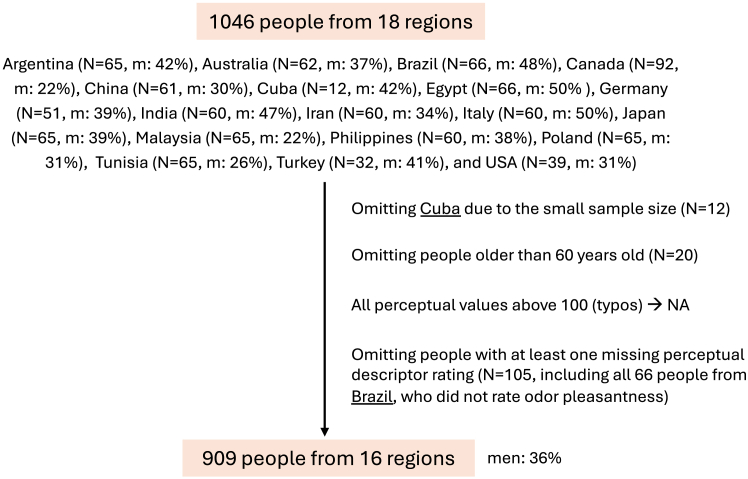
Exclusion criteria and the final sample m, men.

This cross-regional study included 1,046 people from 18 locations. However, people from Cuba were excluded due to the small sample size, and people from Brazil were excluded due to the missing perceptual data (see Figure 2). Therefore, the final sample presented here is composed of 909 people (36% men, 12% smokers) from 16 regions (Argentina, Australia, Canada, China, Egypt, Germany, India, Iran, Italy, Japan, Malaysia, Philippines, Poland, Tunisia, Türkiye, and USA; Table 1; Figure S2). First, the individual descriptor-specific OPFs were calculated as explained in Figure 1. Next, the effect of age, gender, and region on the 12-component descriptor-specific OPFs was evaluated. Permutational multivariate analyses of variance (PERMANOVA) (Table 2) with independent variables age, gender, and region showed that although age and gender significantly affected the OPF, they explained only 1.1% and 0.3% of the variance, respectively. After explaining 1.4% of the variance with age and gender, the region explained further 9.6% of the variance. Yet, the majority of the variance remained unexplained. In addition, another PERMANOVA (Table S1) with independent variables in a different order (region, age, and gender) was performed. Similarly, region, age, and gender explained 10.0%, 0.8%, and 0.3% of the variance, respectively. This indicates that the explained variance by each variable was relatively independent of their sequence and therefore of the variance explained by the other two. Our results suggest that despite age and gender differences between the regions (Table 1), these two variables were of relatively little importance to the OPFs.

**Table 1:** Age and gender per region

Region	CROCUS, *N* (%)	Final sample, *N* (%)	Age,^a^ median (25%–75%)	Gender^a^ (women : men), *N* (%)
All	1046	909	28.0 (23.0–39.0)	578 (64%) : 326 (36%) [5]
Argentina (Buenos Aires)	65 (6%)	56 (6%)	35.5 (27.8–46.0)	29 (55%) : 24 (45%) [3]
Australia (Sydney)	62 (6%)	59 (6%)	20.0 (19.0–27.5)	37 (63%) : 22 (37%)
Brazil (Londrina)^b^	66 (6%)	–	–	–
Canada (Victoria, Trois-Rivieres)	92 (9%)	85 (9%)	24.0 (21.0–36.0)	67 (79%) : 18 (21%)
China (Beijing)	61 (6%)	60 (7%)	26.0 (21.0–45.3)	42 (70%) : 18 (30%)
Cuba (Havana)^c^	12 (1%)	–	–	–
Egypt (Cairo)	66 (6%)	63 (7%)	29.0 (24.0–35.0)	32 (51%) : 31 (49%)
Germany (Dresden)	51 (5%)	51 (6%)	26.0 (22.0–29.0)	30 (60%) : 20 (40%) [1]
India (Pune)	60 (6%)	60 (7%)	26.5 (23.0–39.5)	32 (53%) : 28 (47%)
Iran (Teheran)	60 (6%)	58 (6%)	34.5 (24.3–42.0)	37 (64%) : 21 (36%)
Italy (Cagliari)	60 (6%)	58 (6%)	25.0 (23.0–28.8)	28 (48%) : 30 (52%)
Japan (Tokyo)	65 (6%)	53 (6%)	34.0 (27.0–42.0)	31 (60%) : 21 (40%) [1]
Malaysia (Kuala Lumpur)	65 (6%)	64 (7%)	34.5 (25.0–43.0)	50 (78%) : 14 (22%)
Philippines (Manila)	60 (6%)	59 (6%)	30.0 (27.0–36.0)	36 (61%) : 23 (39%)
Poland (Wroclaw)	65 (6%)	65 (7%)	26.0 (21.0–40.0)	45 (69%) : 20 (31%)
Tunisia (Tunis)	65 (6%)	60 (7%)	29.0 (26.0–38.0)	44 (73%) : 16 (27%)
Türkiye (Izmir)	32 (3%)	27 (3%)	25.0 (19.5–45.0)	17 (63%) : 10 (37%)
USA (Chicago)	39 (4%)	31 (3%)	23.0 (23.0–27.5)	21 (68%) : 10 (32%)
*p* value (effect size)	–	–	<0.001 (eta^2^ = 0.11)	0.003

[], number of missing values; CROCUS, cross-cultural study on variability in chemosensory sensitivity.

a In the final sample.

b Omitted due to the missing odor pleasantness values.

c Omitted due to the small sample size.

**Table 2:** PERMANOVA results with the degrees of freedom, sum of squares, partial *R*^2^, pseudo-*F* statistic, and *p* value

Independent variables	df	Sum of squares	*R*^2^	*F*	*p* value
Age (continuous value)	1	33,727	0.011	11.4	**0.001**
Gender (2 levels)	1	8,472	0.003	2.9	**0.019**
Region (16 levels)	15	283,658	0.096	6.4	**0.001**
Residual	886	2,617,948	0.889	–	–
Total	903^a^	2,943,805	1.000	–	–

Dependent variables were the descriptor-specific OPFs (12 components: appetizing, aromatic, bitter, burnt, disturbing, edible, feminine, intense, medicinal, mouth-odor like, natural, and pleasant).

Independent variables were age (continuous), gender (two levels: women and men), and region (16 levels: Argentina, Australia, Canada, China, Egypt, Germany, India, Iran, Italy, Japan, Malaysia, Philippines, Poland, Tunisia, Türkiye, and USA). Significance for each independent variable was evaluated sequentially from first to last.

df, degrees of freedom.

Significant effects in bold.

a Among people without missing values for gender.

As the analysis of multivariate homogeneity of group dispersions showed significant differences among the regions (Table S2; Figure S3), some part of the explained variance with the region could be due to the difference in dispersion. In other words, people from regions with lower dispersion in their descriptor-specific OPFs (like Germany and Poland) agreed more with each other compared to people from regions with a higher dispersion in their OPFs (like the Philippines). However, this difference in dispersion among the 16 regions was small (*r*^2^ = 0.05).

### Principal-component analysis of the descriptor-specific OPFs

**Figure 3:**
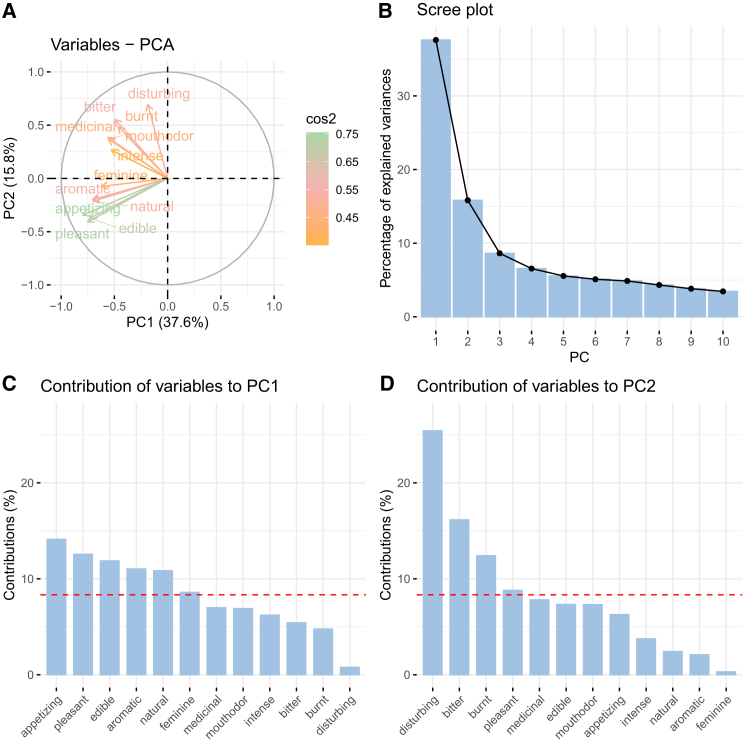
Results from the PCA on the descriptor-specific OPF (A) Plot of the Eigenvectors of variables in PC1 and PC2. (B) A scree plot or a bar graph of the explained variance by each PC. (C and D) Bar graph of the contributions of each OPF descriptor to PC1 and PC2. The red dotted line is the expected average contribution.

**Figure 4:**
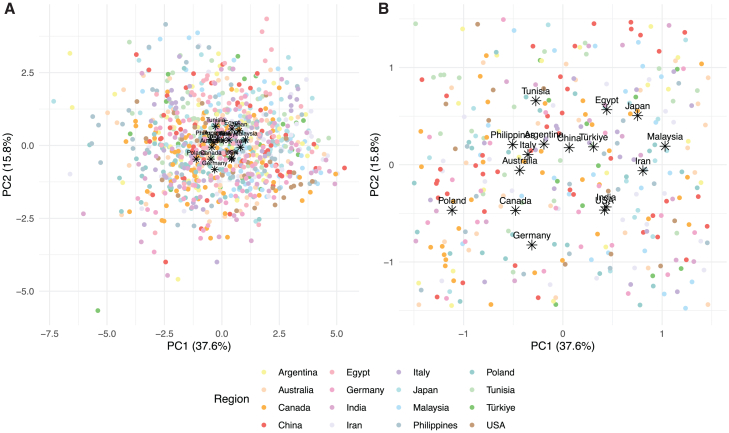
Results from the PCA on the descriptor-specific OPF (A) All individuals are included. (B) The centroids are additionally visualized. Scatterplots of the individual descriptor-specific OPFs plotted on a 2D graph (*x* axis: PC1, *y* axis: PC2).

To visualize the dispersion of the OPFs per region and the region centroids, an unsupervised machine learning method, principal-component analysis (PCA), on the OPFs was performed. The first and the second principal components (PCs) accounted for 38% and 16% of the variance, respectively. Both together explained 54% of the variance (Figure 3). PC1 was mainly loaded by positive OPF descriptors: “appetizing,” “pleasant,” “edible,” “aromatic,” and “natural,” while PC2 was built mainly on OPF descriptors with negative valence: “disturbing,” “bitter,” and “burnt.” The centroids for each region were calculated as the mean PC1 and mean PC2 for each region and visualized on a scatterplot together with the individual descriptor-specific OPFs (Figure 4).

### Region-related differences in the individual OPF descriptors

**Figure 5:**
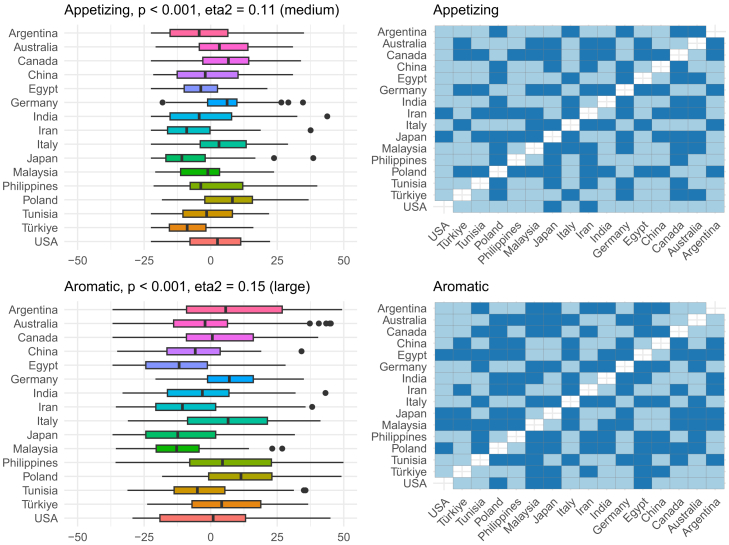
OPF descriptors appetizing and aromatic among the participants (*N* = 909) from the 16 regions Effect size was evaluated using eta squared (eta^2^). On the right, post hoc analysis (Dunn test with Benjamini-Hochberg adjustment for multiple testing) between the regions is shown. Dark and bright blue indicate significant and nonsignificant differences between the two regions, respectively.

**Figure 6:**
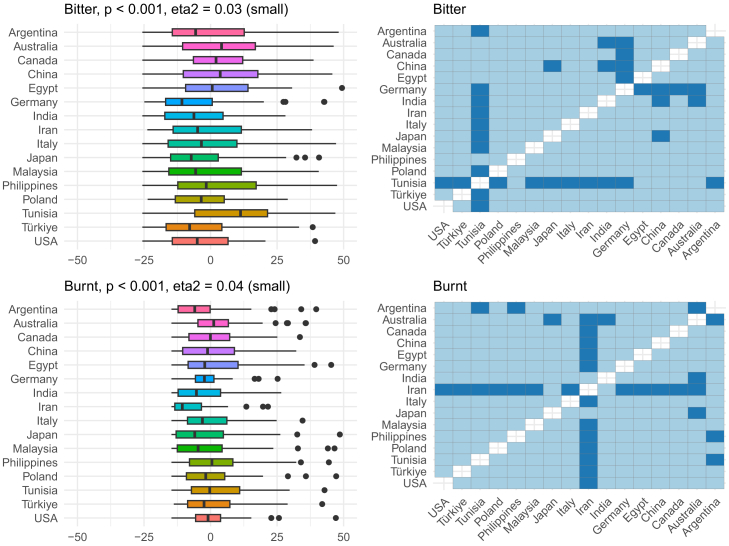
OPF descriptors bitter and burnt among the participants (*N* = 909) from the 16 regions Effect size was evaluated using eta squared (eta^2^). On the right, post hoc analysis (Dunn test with Benjamini-Hochberg adjustment for multiple testing) between the regions is shown. Dark and bright blue indicate significant and nonsignificant differences between the two regions, respectively.

**Figure 7:**
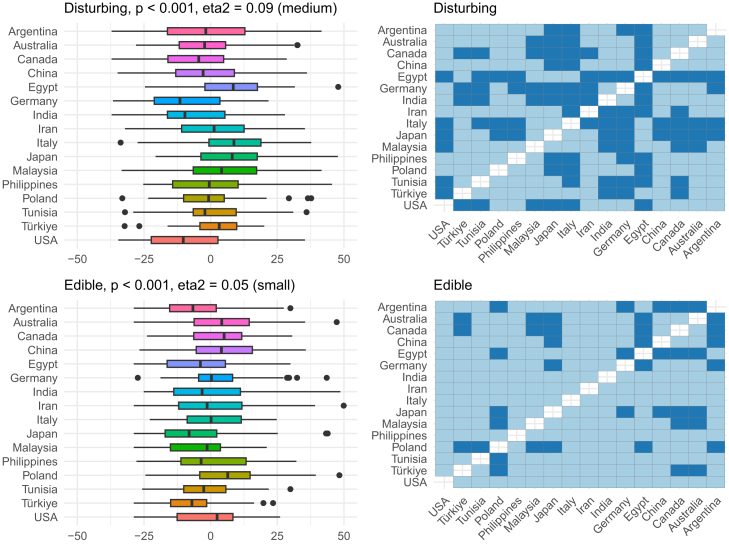
OPF descriptors disturbing and edible among the participants (*N* = 909) from the 16 regions Effect size was evaluated using eta squared (eta^2^). On the right, post hoc analysis (Dunn test with Benjamini-Hochberg adjustment for multiple testing) between the regions is shown. Dark and bright blue indicate significant and nonsignificant differences between the two regions, respectively.

**Figure 8:**
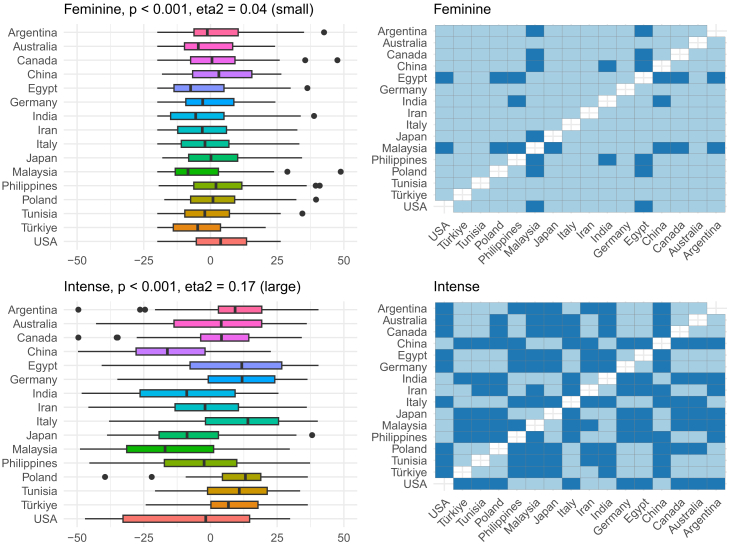
OPF descriptors feminine and intense among the participants (*N* = 909) from the 16 regions Effect size was evaluated using eta squared (eta^2^). On the right, post hoc analysis (Dunn test with Benjamini-Hochberg adjustment for multiple testing) between the regions is shown. Dark and bright blue indicate significant and nonsignificant differences between the two regions, respectively.

**Figure 9:**
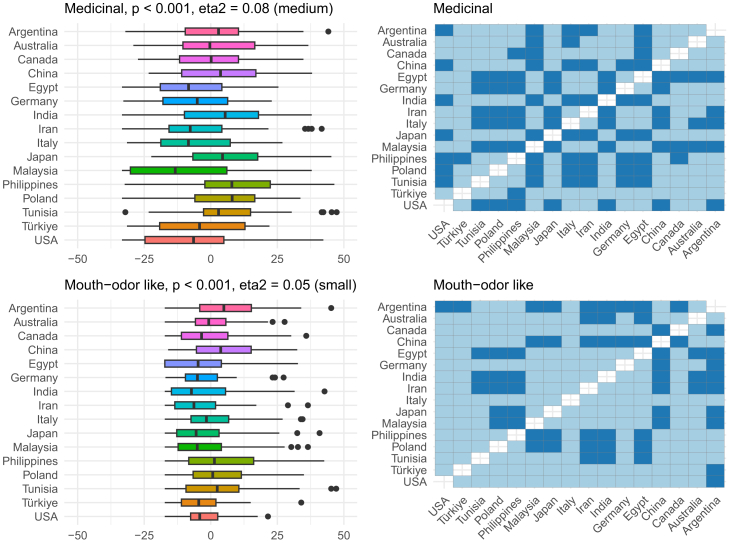
OPF descriptors medicinal and mouth-odor like among the participants (*N* = 909) from the 16 regions Effect size was evaluated using eta squared (eta^2^). On the right, post hoc analysis (Dunn test with Benjamini-Hochberg adjustment for multiple testing) between the regions is shown. Dark and bright blue indicate significant and nonsignificant differences between the two regions, respectively.

**Figure 10:**
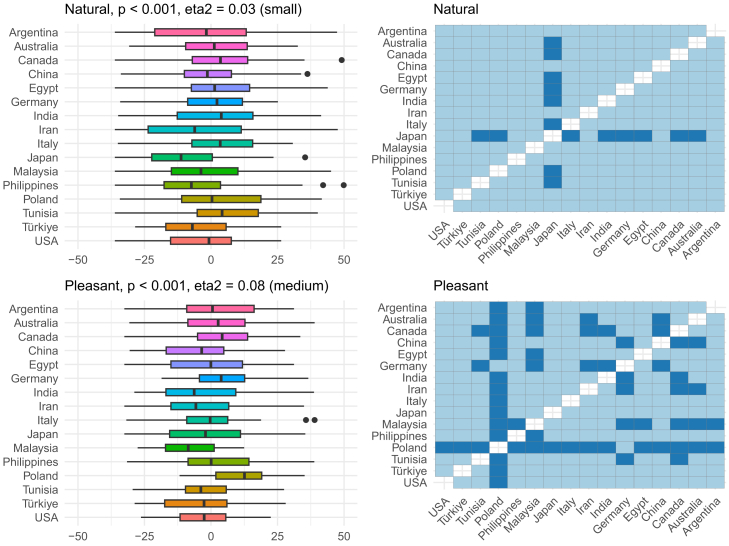
OPF descriptors natural and pleasant among the participants (*N* = 909) from the 16 regions Effect size was evaluated using eta squared (eta^2^). On the right, post hoc analysis (Dunn test with Benjamini-Hochberg adjustment for multiple testing) between the regions is shown. Dark and bright blue indicate significant and nonsignificant differences between the two regions, respectively.

**Figure 11:**
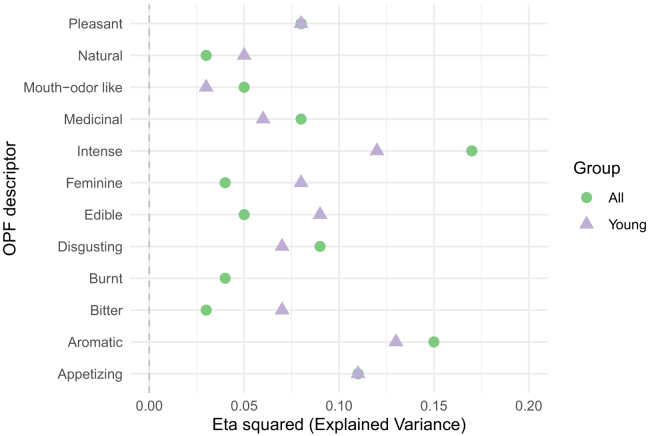
Explained variance in each OPF descriptor on the whole sample (all, *N* = 909) and among people younger than 30 years old (young, *N* = 519) The explained variance for OPF descriptor appetizing and pleasant was the same for both groups.

**Figure 12:**
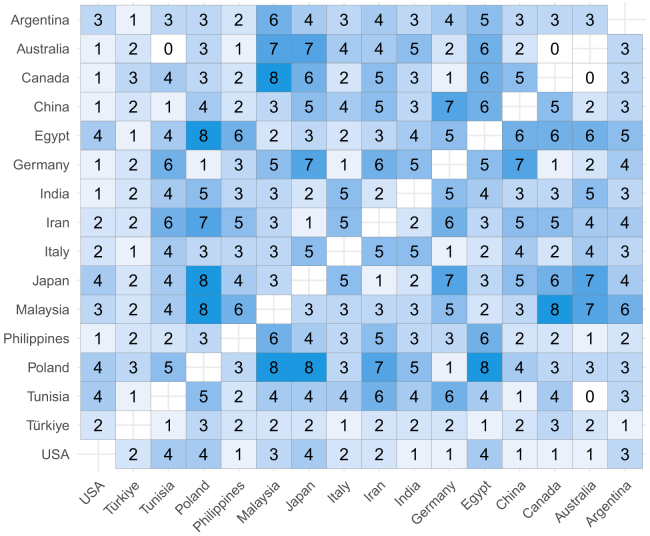
Number of significant differences in the OPF descriptors (e.g., appetizing, aromatic) between the regions Ranging from 0, which indicates that there were no significant differences in the OPF descriptors among the two regions, up to 12, which indicates that the two regions differed in all 12 OPF descriptors.

Overall, the region explained around 10% of the variance in the descriptor-specific OPFs, and this is visualized in Figure 4. To better understand which OPF descriptors were more (or less) region-dependent, further analysis of individual OPF descriptors was performed (Figures 5, 6, 7, 8, 9, and 10). There were significant differences among the 16 regions in all OPF descriptors. The effect size varied from large in cases of aromatic (eta^2^ = 0.15), and intense (eta^2^ = 0.17), medium in cases of appetizing (eta^2^ = 0.11), disgusting (eta^2^ = 0.09), medicinal (eta^2^ = 0.08), and pleasant (eta^2^ = 0.08) to small in the case of bitter (eta^2^ = 0.03), burnt (eta^2^ = 0.04), edible (eta^2^ = 0.05), feminine (eta^2^ = 0.04), mouth-odor like (eta^2^ = 0.05), and natural (eta^2^ = 0.03) (Figures 5, 6, 7, 8, 9, 10, and 11).^23^ To quantify to what extent each region differed in the descriptor-specific OPFs from the other regions, the number of significant differences in the OPF descriptors was plotted for each pair of regions (Figure 12).

### Age and gender-related differences in the individual OPF descriptors

Further analyses showed that gender did not affect the individual OPF descriptors (Figures S4 and S5). On the other hand, most OPF descriptors were negatively correlated with age, yet the correlation was weak (appetizing *p* < 0.001, ρ = −0.14; aromatic *p* = 0.02, ρ = −0.08; bitter *p* < 0.001, ρ = −0.18; burnt *p* < 0.001, ρ = −0.15; edible *p* < 0.001, ρ = −0.16; intense *p* = 0.01, ρ = −0.09; mouth odor-like, *p* < 0.001, ρ = −0.14; natural *p* < 0.001, ρ = −0.12; pleasant *p* < 0.001, ρ = −0.12). Other OPF descriptors were not correlated (*p* > 0.05 for disturbing, feminine, and medicinal). Additional analysis showed that people aged above 50 years perceived odors as less appetizing, less bitter, less burnt, less edible, less natural, and less pleasant compared to people younger than 30 years (Figures S6 and S7). No differences in the individual OPF descriptors were observed among smokers and non-smokers (Figures S8 and S9).

### Results remained similar among people younger than 30 years old

Additionally, the effect of region on the OPF was reanalyzed in people younger than 30 years old (Table S3) to further explore the possible age bias. In line with previous analysis, PERMANOVA including only people younger than 30 years old (*N* = 514), showed that region and gender explained 10.8% and 0.7% of the variance, respectively (Table S4). Of note, the OPF descriptor burnt was not affected by region (*p* = 0.05); otherwise, the overall pattern of results remained similar (Figures S10–S16).

### Results remained similar using the odor-specific OPF

Additionally, the odor-specific OPFs, as suggested by Secundo et al.,^20^ were calculated (Figure S1) to evaluate the effect of geographical and demographic determinants on another measure of olfactory perception. Another PERMANOVA using the odor-specific OPFs (Table 3 for odors) as the dependent variables was performed (Table S5). In line with previous results, age, gender, and region explained 0.5%, 0.3%, and 8.2% of variance in the odor-specific OPFs. Again, the analysis of multivariate homogeneity of group dispersions showed significant differences among the regions (Table S6; Figure S17) with a small effect size (*r*^2^ = 0.04). Additionally, an exploratory analysis, another PCA on the odor-specific OPFs was performed to visualize the dispersion of these OPFs per region and the region centroids (Figures S18 and S19). Of note, the first two PCs accounted for only 41% of the variance; therefore, it has limited relevance. However, it is reported as the visual relations between the regions are somewhat in line with the PCA from the descriptor-specific OPFs (Figure 4).

**Table 3:** Eight odorants with their IUPAC name, CAS reference number, PubChem CID, and quality

*N*	IUPAC name	CAS	CID	Quality (Good Scent Company).^47^
A	ethyl 2-methylbutanoate	7452-79-1	24020	sharp, sweet, green apple, fruity
B	butan-1-ol	71-36-3	263	cheesy, old socks, body odor
C	2-methoxyphenol	90-05-1	460	smoky, spicy, medicinal, glue, savory, meaty, woody
D	1-methoxy-4-[(E)-prop-1-enyl]benzene	4180-23-8	637563	sweet, anise, licorice, medicinal
E	2-methoxy-4-prop-2-enylphenol	97-53-0	3314	sweet, spicy, clove, woody, ham, bacon, cinnamon, allspice
F	3-methylbutyl acetate	123-92-2	31276	sweet, banana, fruity, ripe
G	(*Z*)-hex-3-en-1-ol	928-96-1	5281167	fresh, grassy, herbal oily
H	3-methylbutanoic acid	503-74-2	10430	sour, sweaty, cheesy, tropical

CAS, chemical abstract service; CID, compound identification.

## Discussion

This cross-regional study, including 909 people from 16 regions, aimed to evaluate how region influences human olfactory perception as described by the OPFs. When evaluating the descriptor-specific OPFs, region, age, and gender explained around 9.6%, 1.1%, and 0.3% of the variance, respectively. Yet, the majority of the variance remained unexplained. These findings were also observed in a subsample of the participants who were younger than 30 years. Two PCs emerged from the 12 descriptors used, loaded mainly by (1) positive and (2) negative-valenced descriptors. Further analysis of individual OPF descriptors showed that region explained from 3% (bitter and natural) up to 17% (intense) of the variance in a single OPF descriptor. Importantly, results remained similar using another measure of the olfactory perception, the odor-specific OPFs. More precisely, age, gender, and region explained 0.5%, 0.3%, and 8.2% of variance in the odor-specific OPFs. Again, leaving the majority of variance unexplained.

To our knowledge, this is the first study to evaluate descriptor-specific OPFs among healthy people inhabiting different regions of the globe with different cultural influences. When comparing our results to previous studies, one must keep in mind that an individual OPF descriptor describes a perceptual dimension across multiple odors, while previous studies mostly compared the perception of individual odors and were often limited to hedonic perception.^13^^,^^14^^,^^15^ Odor-specific differences in odor pleasantness and intensity among various regions have been shown.^5^^,^^14^^,^^16^^,^^17^^,^^24^ Culture explained 6%–7% of the variance in odor pleasantness ratings,^13^^,^^14^^,^^15^ and here, 8% of the variance of the pleasantness component of the descriptor-specific OPF was explained by region.

Interestingly, the OPF descriptor “odor intensity” was twice as discriminative as “odor pleasantness” (17% vs. 8%). Cross-cultural differences in odor intensity have been reported before,^5^^,^^16^^,^^17^ suggesting that the region people inhabit influences more what they find intense rather than what they find pleasant. Our study supports the notion that human olfactory perception, although likely driven in part by hedonic aspects, may be better differentiated by odor intensity. Considering this, more studies on individual and cultural determinants of intensity perception should be pursued.

In this study, region influenced odor perception to a greater extent than age or gender. In other words, people from the same region were more similar to each other regardless of their age and gender compared to people from other regions. Indicating that the cultural consistency in odor perception is something that develops already at a young age. Of note, only people younger than 60 years were included in this study, reducing the effect of age-related olfactory dysfunction,^25^^,^^26^ which has been shown to alter one’s olfactory perception and the OPFs.^21^^,^^22^ Age is considered a key factor for olfactory performance^27^^,^^28^; however, in this study, we were interested in the qualitative perception of odors rather than the (in)ability to smell. With the age limit of 60 years, we can assume that our sample was not largely affected by the smell deficits resulting from aging.^25^^,^^28^

We also did not observe a major role of gender in explaining variability in the OPFs. While women may score higher in the smell tests evaluating their olfactory function (e.g., odor threshold, discrimination, and identification),^29^ their qualitative perception of odors seems relatively similar to men’s across various locations. The relatively low importance of gender in the OPFs is still intriguing given that most explanations of the difference in olfactory awareness and importance of olfaction refer to differential expertise and use of odors in daily life (i.e., women can be more experienced with odors because they cook, buy cosmetics). More studies focused on the role of gender in shaping olfactory perception is required.

The majority of the variance in the descriptor-specific and odor-specific OPFs remained unexplained with region, age, and gender. Thus, confirming that individual olfactory perception is a complex and multi-faceted phenomenon. While some of the unexplained variance could be noise, another part could be due to other individual-related factors such as olfactory receptor expression differences,^30^^,^^31^ physiological state,^6^ personality,^32^ attention,^33^ olfactory (dys)function,^21^^,^^22^ diseases,^34^ or uncontrolled context-related factors such as air temperature, humidity^35^ (but see Drews et al.^36^), exposure to ambient air odors,^37^^,^^38^^,^^39^ and many other factors.

Importantly, descriptor-specific OPF and odor-specific OPF yielded congruent results, and both left the majority of variance unexplained. Although the odor-specific OPF was shown to be related to individual genetic materials,^20^ the descriptor-specific OPF has not been evaluated in this way.^19^ We suggest that the OPF method could alternatively be termed “olfactory perceptual dimensions.” Further studies comparing the two approaches are needed to calibrate the terminology.

To conclude, 10% of the variance in the descriptor-specific OPFs and 8% in the odor-specific OPF was explained by region, with some of these effects attributable to dispersion. Thus, it remains ambiguous to what extent culture influences odor perception and to what extent certain regions exhibit greater heterogeneity in odor responses. Additionally, from 0.3% to 1.1% of the variance was explained by age and gender, yet the majority of variance remained unexplained. Interestingly, odor intensity was more region-dependent than odor pleasantness. Our findings indicate that region-related olfactory experiences, to some extent, influence human olfactory perception, but, overall, humans perceive odors quite similarly in different regions of the world.

### Limitations of the study

The current study presented evidence based on a large sample of 909 individuals who have been tested psychophysically in personal contact with an experienced researcher. One limitation is that data collection was mostly performed in a single region of a given country (with the exception of Canada, where we tested subjects in two distinct locations). Results from a given location are not representative of a whole country, knowing that differences in olfactory perception exist also within a country.^40^^,^^41^^,^^42^ The current study describes olfactory perception of individuals inhabiting urban areas, whereas it does not tackle the perception of people living in rural areas, likely characterized by different olfactory experiences and olfactory landscape. This is, however, difficult to counteract, as the olfactory laboratories or clinics where the measurements were performed are often located in urban areas. Furthermore, our sample was gender imbalanced. While worth mentioning, we note that our regional samples do not mean to be representative of the population. The greater representation of women likely results from their greater interest in odors and the importance of olfaction than men.^43^^,^^44^ Thus, women may have more eagerly enrolled for the olfaction-oriented study. Furthermore, we believe that this did not greatly influence the overall results due to the small gender effect on the OPFs, which remained stable in the PERMANOVA analysis regardless of the order of the independent variables. Additionally, although the two ways of evaluating olfactory perception (i.e., OPFs) provided congruent results, one must keep in mind their caveats. The descriptor-specific OPF^19^ is odor-independent; however, it is strongly influenced by the perceptual descriptors, and its results might, to some extent, differ when using different ones. On the other hand, the odor-specific OPF^20^ is odor-dependent, and its results might differ when using different odors. Both multi-dimensional olfactory perceptual frameworks require systematic empirical testing. Another limitation of this study is that odors were evaluated only once, although individual perception might differ as a function of time. While decent temporal stability of the odor-specific OPF was shown,^20^ this has to be further evaluated for the descriptor-specific OPF.^19^

## Resource availability

### Lead contact

Further information and requests for resources should be directed to and will be fulfilled by the lead contact, Anna Oleszkiewicz (ania.oleszkiewicz@gmail.com).

### Materials availability

This study did not generate new unique reagents.

### Data and code availability

• All data have been deposited at Open Science Framework: https://osf.io/m95z4 and are publicly available as of the date of publication.
• All original code has been deposited at Open Science Framework: https://osf.io/m95z4 and is publicly available as of the date of publication.
• Any additional information required to reanalyze the data reported in this article is available from the lead contact upon request.

## Acknowledgments

A.O. was supported by the National Science Center (Poland) grant OPUS 20 #2020/39/B/HS6/01533. E.D. and T.H. were supported by Volkswagen Stiftung (Germany) grant Az 96632, Olfactorial Perceptronics.

## Author contributions

Conceptualization, A.O. and T.H.; methodology, A.O. and T.H.; project administration, A.O. and T.H.; investigation, all authors; formal analysis, D.M., B.Z., and E.D.; visualization, E.D. and A.O.; supervision, A.O. and T.H.; writing – original draft, E.D. and A.O.; writing – review & editing, all authors.

## Declaration of interests

Since 2021, T.H. has collaborated on research projects with Sony, Stuttgart, Germany; Smell and Taste Lab, Geneva, Switzerland; Takasago, Paris, France; and aspuraclip, Berlin, Germany. He received consultant fees from Baia Foods, Madrid, Spain; Burghart, Holm, Germany; and air-up, Munich, Germany. None of these companies were financially involved in this study or had influenced its conceptualization, design, data analysis, or manuscript preparation.

## STAR★Methods

### Key resources table

**Table:** 

REAGENT OR RESOURCE	SOURCE	IDENTIFIER
**Deposited data**		

The collected dataset is available at Open Science Framework: https://osf.io/m95z4/	N/A	https://osf.io/m95z4/

**Experimental models: Organisms/strains**		

Human adults	Recruited in 18 different countries	

**Software and algorithms**		

The code to analyze the data is available at Open Science Framework: https://osf.io/m95z4/	N/A	https://osf.io/m95z4/

### Experimental model and study participant details

#### Human participants

The local teams in 18 countries were asked to recruite participants from the academic (50%) and the general population (50%). Subjects were expected to inhabit the city where the study was executed for at least six months.^45^ The expected age ranged from 18 to 60 years when olfactory functions are optimal.^25^ Further exclusion criteria were self-declared major sense of smell abnormalities, abnormal trigeminal sensitivity to stinging and burning odors like vinegar, major health problems, acute or pronounced chronic inflammation of the mouth or nose and nasal sinuses, and pregnancy.

Overall, the project included 1046 people from 18 countries (Argentina [Buenos Aires], Australia [Sydney], Brazil [Londrina], Canada [Victoria, British Columbia; Trois-Rivieres, Quebec], China [Beijing], Egypt [Cairo], Cuba [Havana], Germany [Dresden], India [Pune], Iran [Teheran], Italy [Cagliari], Japan [Tokyo], Malaysia [Kuala Lumpur], Philippines [Manila], Poland [Wroclaw], Tunisia [Tunis], Türkiye [Izmir], and USA [Chicago]). However, people from Cuba and Brazil were excluded due to the small sample size and missing perceptual data, respectively (Figure 2). The final sample presented here is composed of 909 participants (36% men) aged 18-60 years (median [interquartile range] 28.0 [23.0 – 39.0]) from 16 countries. Details can be found in Table 1.

The study was performed in accordance with the Declaration of Helsinki on Biomedical Studies Involving Human Subjects. Written informed consent was obtained from all participants. The entire study design and consent approach were approved by the Ethics Review Board at the University of Wroclaw (3/2021) and the Institute of Psychology (2021/RYHNA) and TU Dresden (BO-EK-70022023). Furthermore, wherever necessary, additional local ethical approvals were obtained by the local teams.

### Method details

To execute this project, a network of scientific collaborators was established under the CROss-CUltural Study on Variability in Chemosensory Sensitivity (CROCUS) project. Local teams were recruited for this project via a personal network, based on their expertise, and formerly received training at the Smell and Taste Clinic, Dept. of Otorhinolaryngology, TU Dresden. The final sample comprised 18 local teams in Argentina (Buenos Aires), Australia (Sydney), Brazil (Londrina), Canada (Victoria, British Columbia; Trois-Rivieres, Quebec), China (Beijing), Egypt (Cairo), Cuba (Havana), Germany (Dresden), India (Pune), Iran (Teheran), Italy (Cagliari), Japan (Tokyo), Malaysia (Kuala Lumpur), Philippines (Manila), Poland (Wroclaw), Tunisia (Tunis), Türkiye (Izmir), and USA (Chicago). Beyond a common training at the Smell and Taste Clinic, TU Dresden, study coherence was ascertained by several other actions taken by the coordinators of the project. All local teams received documents describing the procedure along with a video recording of each step of the procedure and score calculation. Before the study commencement, two Zoom conferences were scheduled for the coordinators to respond to all questions raised by the local teams. The local teams back-translated the protocol, and the participants were tested in their native language. If there were doubts about the individual descriptor back-translations, AO resolved them with the local teams. All materials were manufactured by Burghart Messtechnik (Holm, Germany) and shipped to the local teams. All participants within each location were tested in ventilated rooms, during individual sessions (approximately 60 min). We refer to the testing site/country as ‘region’ to avoid stretching conclusions to the entire country while the data were collected in a given city within a country. We understand region as a proxy for the individual experience of odors resulting from varying cultural practices and local physical conditions (e.g., atmospheric).

#### Procedure

First, sociodemographic data such as age, gender, and residency were collected via a brief interview. Next, participants received eight odorous felt-tip pens (so-called Sniffin’ Sticks^46^) marked from 1 to 8. They were asked to smell and rate each odor on a scale from 0 (»minimum value of the descriptor«) to 100 (»maximum value of the descriptor«) on a 100mm long visual analog scale (VAS). For each odor, there were 12 VAS scales, one for each of the 12 perceptual descriptors: appetizing, aromatic, bitter, burnt, disturbing, edible, feminine, intense, medicinal, mouth odor, natural, and pleasant.^20^ Participants could smell each odor as many times as they wished. To ease the test administration and standardize the display, the scores marked on VAS were collected paper-and-pencil and further transformed into a digital score by measuring the distance between 0 (beginning of the VAS scale) to the spot where the answer of the participant crossed the VAS scale (in mm).

#### Descriptor-specific Olfactory Perceptual Fingerprint

For the perceptual odor ratings, eight odors were chosen inspired by Secundo et al.^20^ (Table 3) and modified according to the market availability. The individual descriptor-specific OPFs were calculated as Snitz et al. suggested (Figure 1).^19^ Each participant m rated M (M=8) odors using N (N=12) perceptual descriptors, resulting in 96 individual data points for odor ratings. For the descriptor-specific OPF, the difference between the individual rating for odor i using descriptor j versus the entire sample average rating for odor i using descriptor j was calculated. After that, each individual was described using an M ∗ N matrix of relative scores for each descriptor and each odor. Then, M relative scores were averaged along each of the descriptors N. In the end, the twelve-dimensional vector, the OPF, described their olfactory perception.

#### Odor-specific olfactory perceptual fingerprint

Additionally, the odor-specific OPFs as suggested by Secundo et al.^20^ were calculated. Here, Euclidean distances between all pairs of odors were calculated as shown on Figure S1. Since participants rated eight odors, this OPF was composed of 8x7/2 = 28 components, which represent all the pairwise odor combinations. When comparing our calculation with the one by Secundo et al.,^20^ we omitted the division by √n (n is the number of perceptual descriptors used to rate each odor), because there were no missing values in our dataset, therefore the normalization of the data was unnecessary. Of note, the reported results remain the same even if all pairwise distances are divided by √12.

### Quantification and statistical analysis

The median and interquartile range were used to describe the continuous variable’s central tendency and variance such as age. Mann-Whitney test was used to compare non-normally distributed continuous variables. Frequencies were used to describe the distribution of categorical variables, and χ2 tests were used to compare categorical variables, such as gender.

To evaluate whether the descriptor-specific OPFs were affected by the age and gender differences among the sixteen regions and whether the OPFs were region-dependent, two permutational multivariate analyses of variance (PERMANOVA) were performed. The dependent variables were the descriptor-specific OPFs. In the first PERMANOVA, independent variables were age, gender, and region. In the second PERMANOVA, independent variables were region, age, and gender. Both analyses were performed using the option “*by = terms*” to assess the significance of each independent variable sequentially from first to last. PERMANOVA with 999 permutations was applied on the Euclidean distance matrix of the OPFs using an *adonis2* function from the *vegan* package.^48^

To check whether the groups of the independent variables differed in their dispersions, the PERMDISP2 procedure for the analysis of multivariate homogeneity of group dispersions (variances) was performed using a *betadisper* function from the *vegan* package.^48^ As group dispersions of the 16 regions differed significantly, the post hoc analysis using Tukey's ‘Honest Significant Difference’ (HSD) was performed. To better evaluate the dispersion effect, the distances to the centroid per region were visualized using *ggplot2* from the *tidyverse.*^49^

To explore the OPFs and the centroids for each region, principal component analysis (PCA), an unsupervised machine learning method for dimensionality reduction, was performed. Firstly, a function *prcomp* on scaled data was performed. Next, the centroids for each region were calculated as the mean of the first principal component (PC) and the mean of the second PC for each region and visualized on a scatterplot. Loading scores or contributions of variables to the PCs were visualized using a *factoextra* package.^50^

To further evaluate the effect of region on individual OPF descriptors, Kruskal–Wallis analysis of variance was used. The effect size for Kruskal–Wallis was evaluated with an eta-squared (eta^2^) estimate using an R package *rstatix.*^23^ Values from 0.01 to 0.06 indicated a small effect size, from 0.06 to 0.14 indicated a moderate effect size and values above 0.14 indicated a large effect size.^23^ For post hoc analysis, the Dunn test with Benjamini & Hochberg adjustment for multiple testing was performed using the *FSA* package.^51^ To better evaluate how much odor perception differed among two particular regions, the number of differences in the OPF descriptors between the two regions was counted.

To estimate the effect of age, gender, and smoking on individual OPF descriptors, Mann-Whitney test was used. People younger than 30 years old were compared to people older than 50 years old. Additionally, Vargha and Delaney’s A was calculated as an effect size measure using an R package *effsize*. Small effect size was indicated by values from 0.56 to 0.64 and from 0.34 to 0.44, medium effect size was indicated from 0.64 to 0.71 and from 0.29 to 0.34, and large effect size was indicated by values above 0.71 and below 0.29.^52^ Additionally, Spearman correlation coefficients were used for assessing the age effect on individual OPF descriptors. Small effect size was indicated by ρ values from 0.1 to 0.3, medium effect size was indicated by ρ values from 0.3 to 0.5, and large effect size was indicated by values larger than 0.5.^53^ For clarity, the effect sizes references are gathered also in Table S7.

A p-value of < 0.05 was considered statistically significant. All statistical analyses were performed using R Statistical Software^54^ (version 4.2.2; R Foundation for Statistical Computing, Vienna, Austria) with an additional package for data manipulation *tidyverse*^49^ and data visualization *ggpubr.*^55^
