# Supplementary material for: Demographic and geographical determinants of human olfactory perception of 909 individuals inhabiting 16 regions
Source: iScience. 2025 Sep 18;28(10):113455. doi: 10.1016/j.isci.2025.113455 (PMC12570350; doi:10.1016/j.isci.2025.113455)
Supplement: Document S1. Figures S1–S19 and Tables S1–S7 [file mmc1.pdf]

iScience, Volume *28*

Supplemental information

Demographic and geographical determinants of human olfactory perception of 909 individuals inhabiting 16 regions

Eva Drnovsek, Nixon M. Abraham, Jancy N. Abraham, Rafieh Alizadeh, Ines Aloulou, Lixin Chen, Ma. Lourdes Berioso Enecilla, Marco Aurélio Fornazieri, Johannes Frasnelli, Juan Martin Fuselli, Fatima Gansatao, Cagdas Guducu, Anna Kristina Hernandez, Marlise K. Hofer, Salina Husain, Reda Kamel, Vitoria F. Khouri, Francesco Loy, Mehmet K. Mahmut, Daniel Marek, Carla Masala, Natália Medeiros Dias Lopes, Élizabeth Michaluk, Imen Miri, Marjan Mirsalehi, Plamena Miteva, Anasuha Musa, Hanène Naija, Keigo Nakaachi, Michal Pieniak, Jayant M. Pinto, Patricia Portillo Mazal, Ahmed Radwan, Farhad Rafiei, Devesh Rawat, Aleksandra Reichert, Henrique O. Scussiatto, Hozifa Alsaid Sheta, Sharanya M. Thodupunoori, Brianna J. Turner, Hangying Wu, Fiona Wylie, Ayaho Yoshino, Laiquan Zou, Barbara Zyzelewicz, Gregory N. Bratman, Asifa Majid, Thomas Hummel, and Anna Oleszkiewicz

Figure S1. The Odor-Specific Olfactory Perceptual Fingerprint (OPF) for one participant[S1], related to the Introduction and the "Odor-Specific Olfactory Perceptual Fingerprint" section of the STAR Methods.

ODOR	appetizing	aromatic	bitter	burnt	disturbing	edible	feminine	intense	medicinal	mouth-odor like	natural	pleasant
**A**	91	100	2	2	7	92	83	100	76	0	90	92
**B**	1	1	88	91	91	1	0	100	37	54	2	2
**C**	3	91	3	91	92	2	2	93	50	3	2	2
**D**	4	93	3	91	52	1	1	91	91	2	3	63
**E**	49	90	7	53	41	5	5	91	54	4	64	53
**F**	68	87	6	4	4	82	82	55	5	3	87	55
**G**	38	35	3	39	35	33	3	39	39	3	83	28
**H**	1	91	73	82	98	2	1	92	72	72	2	2

$$distance_{k,m} = \sqrt{\sum_{i=1}^{n} (p_i^k - p_i^m)^2}$$

$$distance_{odor\ A,\ odor\ B} = \sqrt{(91-1)^2 + (100-1)^2 + \ldots + (92-2)^2} = 275.2$$

Odor-Specific OPF (Secundo et al.) = [AB, AC, AD,, ..., GH]

Odor-Specific OPF (Secundo et al.) = [275.2, 232.8, 203.2, ..., 179.12]

Perceptual ratings by one individual for the 8 odors (from A to H) using the 12 perceptual descriptors are reported. Below is the equation for each pairwise odor distance (Euclidean distance) with an example for the distance between odor A and B for this individual. Additionally, an example of the Odor-Specific OPF[S1] in general and for this individual is shown. Since participants rated 8 odors (from A to H), this Odor-Specific OPF was composed of 8x7/2 = 28 components, which represent all the pairwise odor combinations.

p_i^k is the perceptual rating of odorant k using descriptor i;

p_i^m is the perceptual rating of odorant m using descriptor i

Figure S2. Boxplots of age per region, related to the “Region-related differences explained 10% of variance in the Descriptor-Specific OPFs” section of the results.

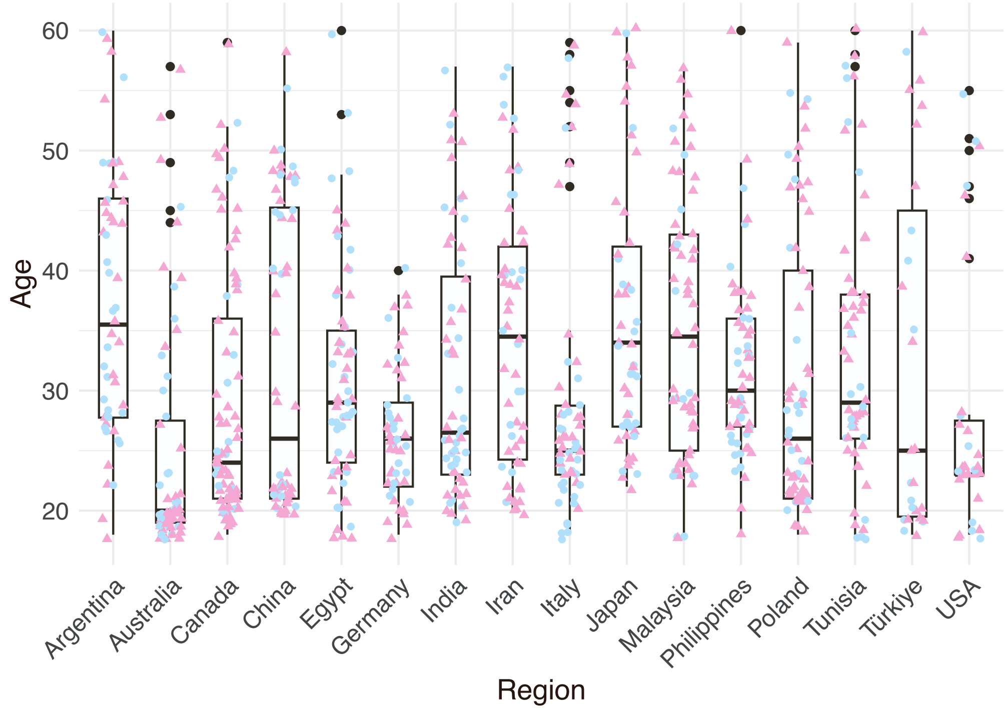

Pink triangles are women, blue dots are men.

Figure S3. Boxplots showing the distances to the centroid by region calculated using the PERMDISP2 procedure for the analysis of multivariate homogeneity of group dispersions (variances) for the 16 regions, related to the "Region-related differences explained 10% of variance in the Descriptor-Specific OPFs" section of the results.

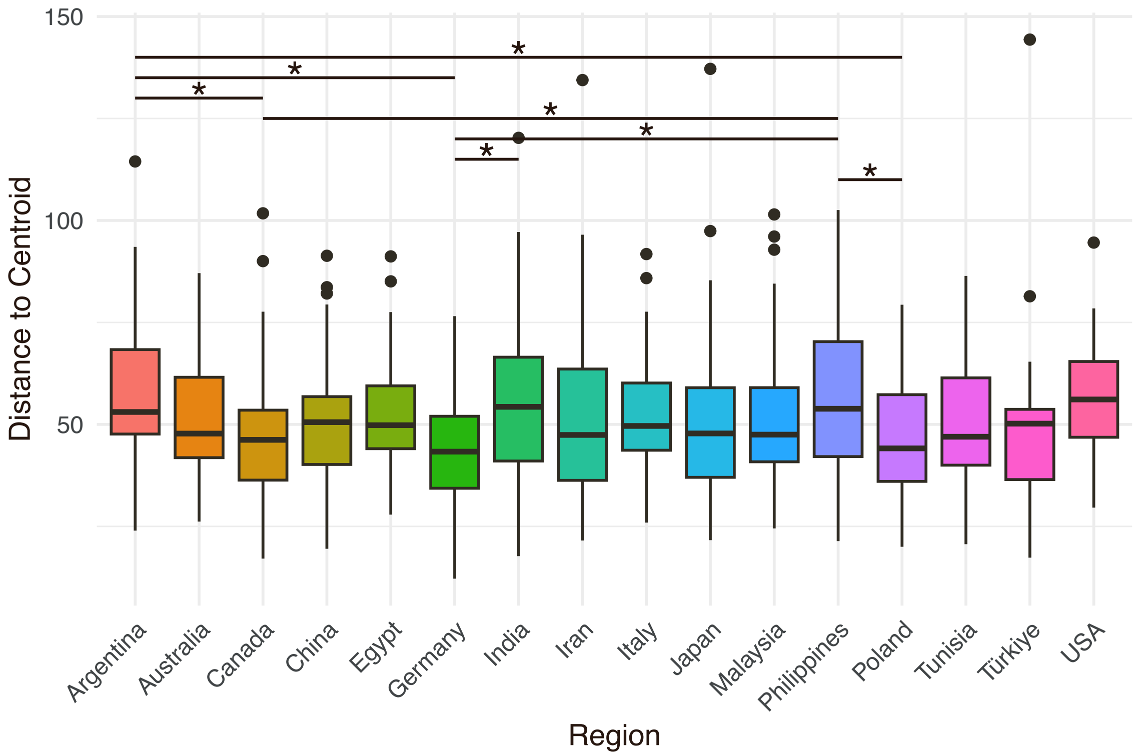

The asterisks indicate the significant differences from the post hoc analysis.

Figure S4. Olfactory Perceptual Fingerprint descriptors appetizing, aromatic, bitter, burnt, disturbing, and edible among men (N = 326) and women (N = 578), related to the "Age and gender-related differences in the individual OPF descriptors" section of the results.

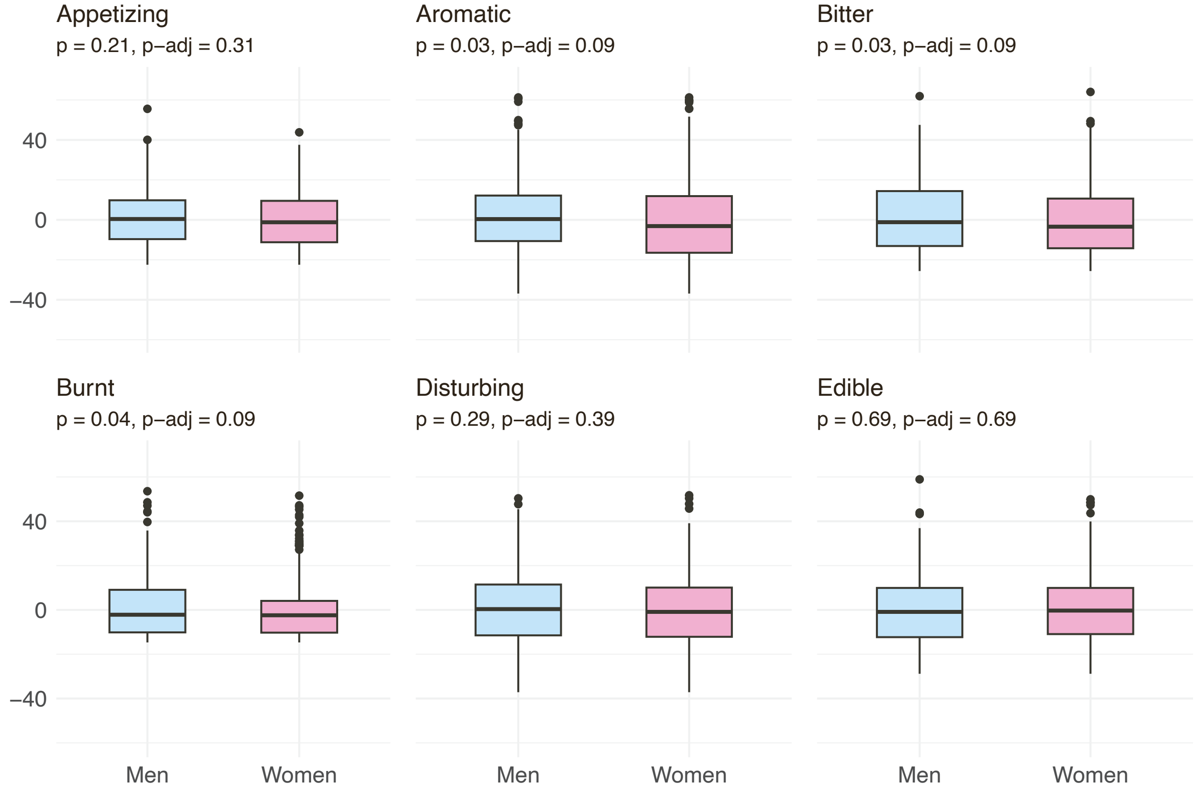

P values were adjusted using a Benjamini & Hochberg adjustment method for multiple testing.

Figure S5. Olfactory Perceptual Fingerprint descriptors feminine, intense, medicinal, mouth odor, natural, and pleasant among men (N = 326) and women (N = 578), related to the “Age and gender-related differences in the individual OPF descriptors” section of the results.

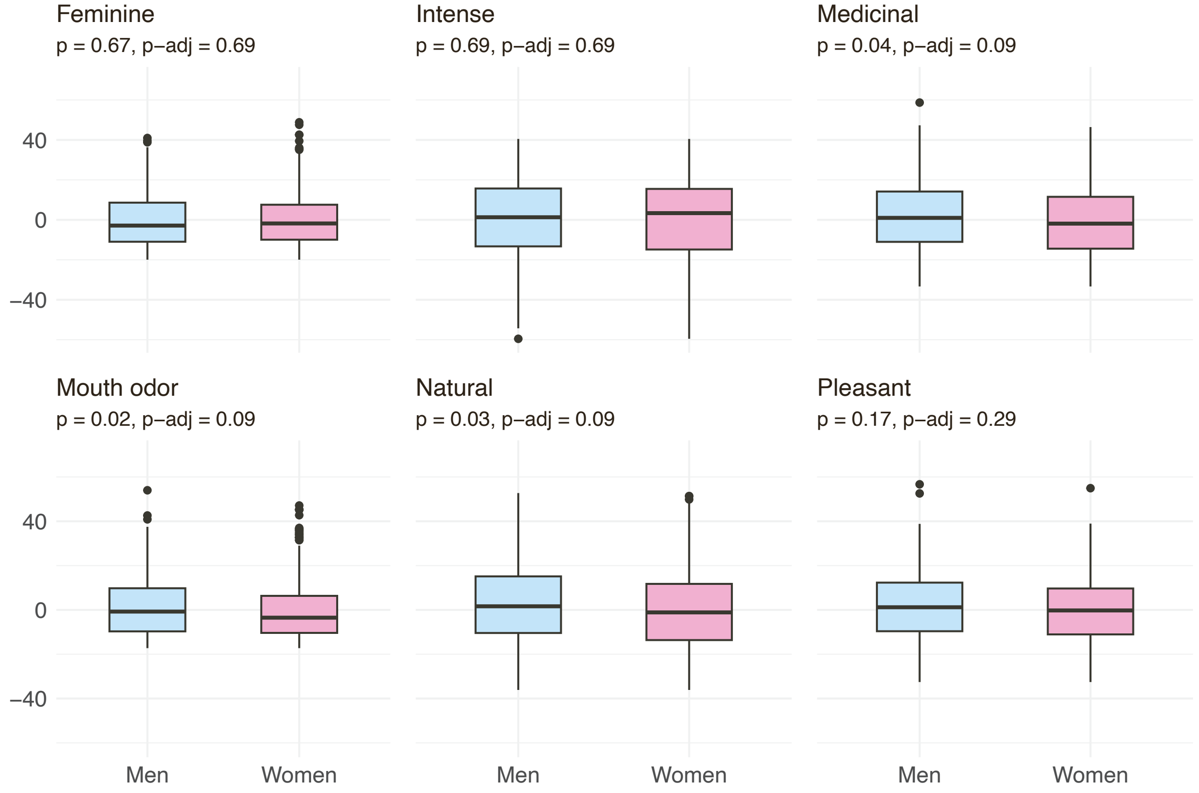

P values were adjusted using a Benjamini & Hochberg adjustment method for multiple testing.

Figure S6. Olfactory Perceptual Fingerprint descriptors appetizing, aromatic, bitter, burnt, disturbing, and edible among people younger than 30 years old (N = 519) and people older than 50 years old (N = 79), related to the "Age and gender-related differences in the individual OPF descriptors" section of the results.

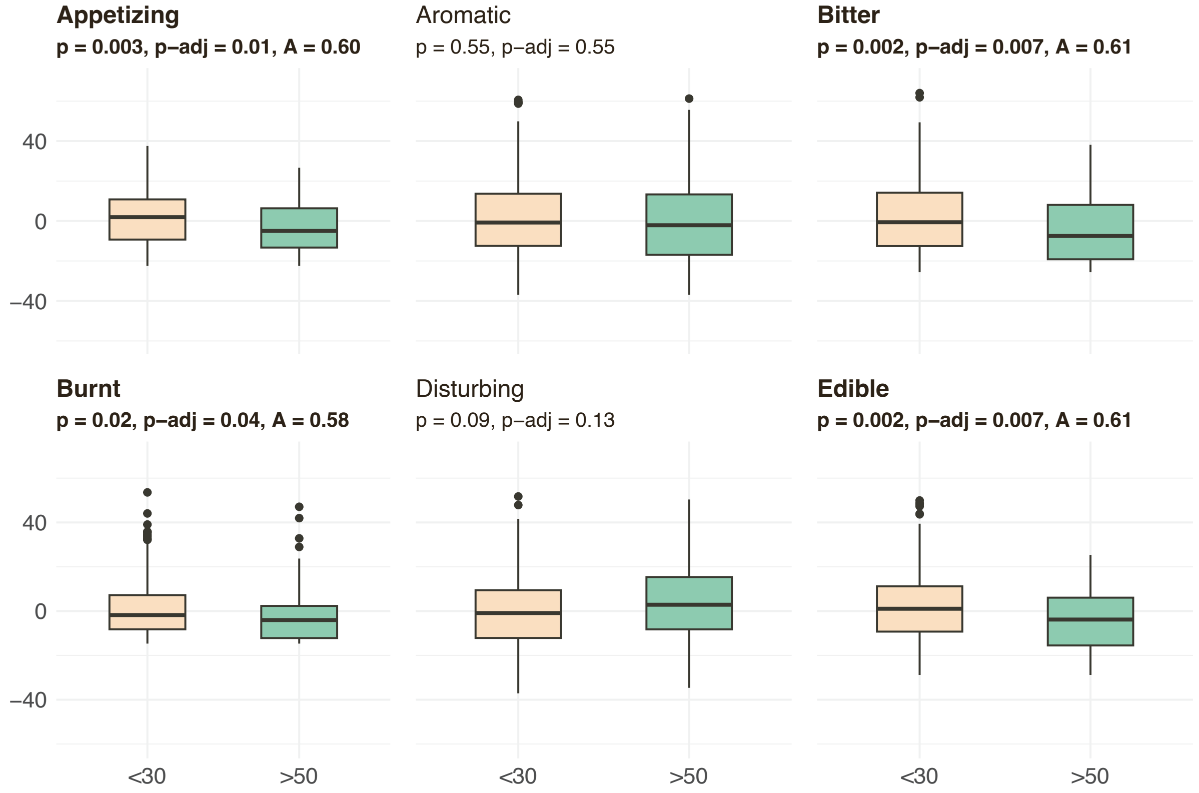

P values were adjusted using a Benjamini & Hochberg adjustment method for multiple testing. Vargha and Delaney's A effect size is reported.

Figure S7. Olfactory Perceptual Fingerprint descriptors feminine, intense, medicinal, mouth odor, natural, and pleasant among people younger than 30 years old (N = 519) and people older than 50 years old (N = 79), related to the “Age and gender-related differences in the individual OPF descriptors” section of the results.

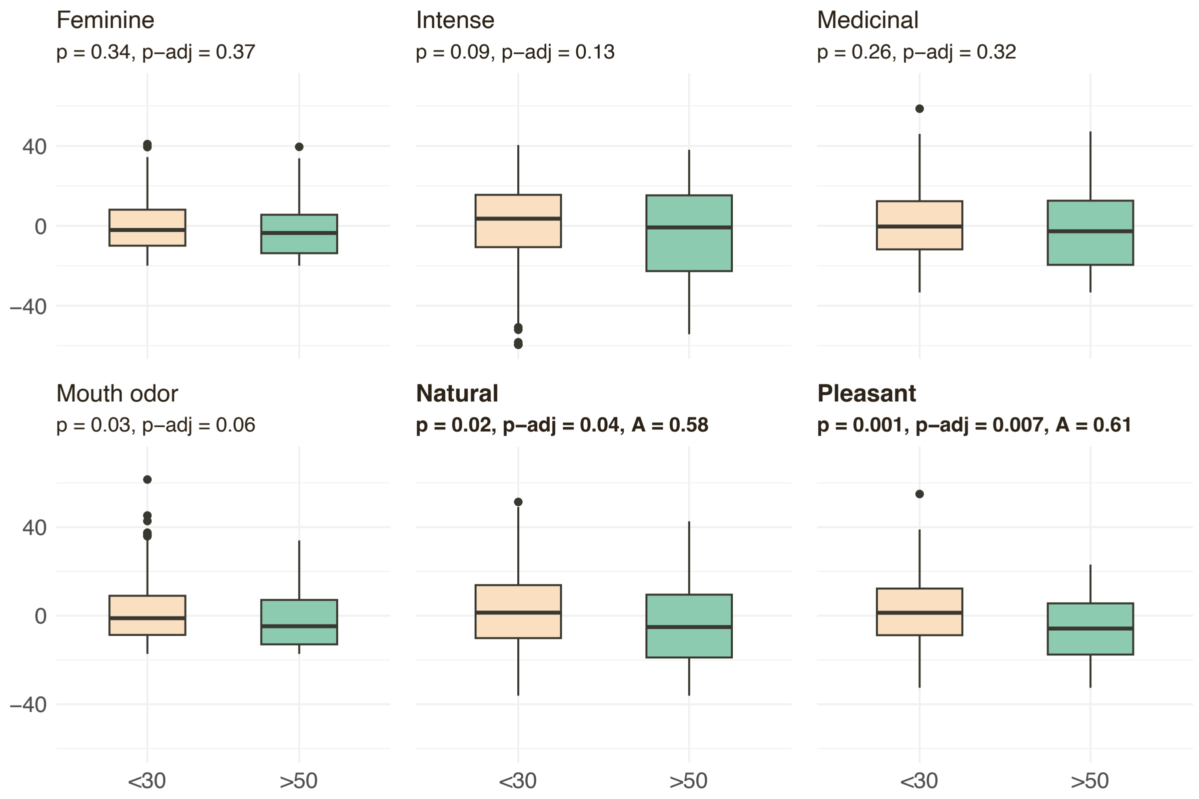

P values were adjusted using a Benjamini & Hochberg adjustment method for multiple testing. Vargha and Delaney’s A effect size is reported.

Figure S8. Olfactory Perceptual Fingerprint descriptors appetizing, aromatic, bitter, burnt, disturbing, and edible among non-smokers (N = 796) and smokers (N = 111), related to the “Age and gender-related differences in the individual OPF descriptors” section of the results.

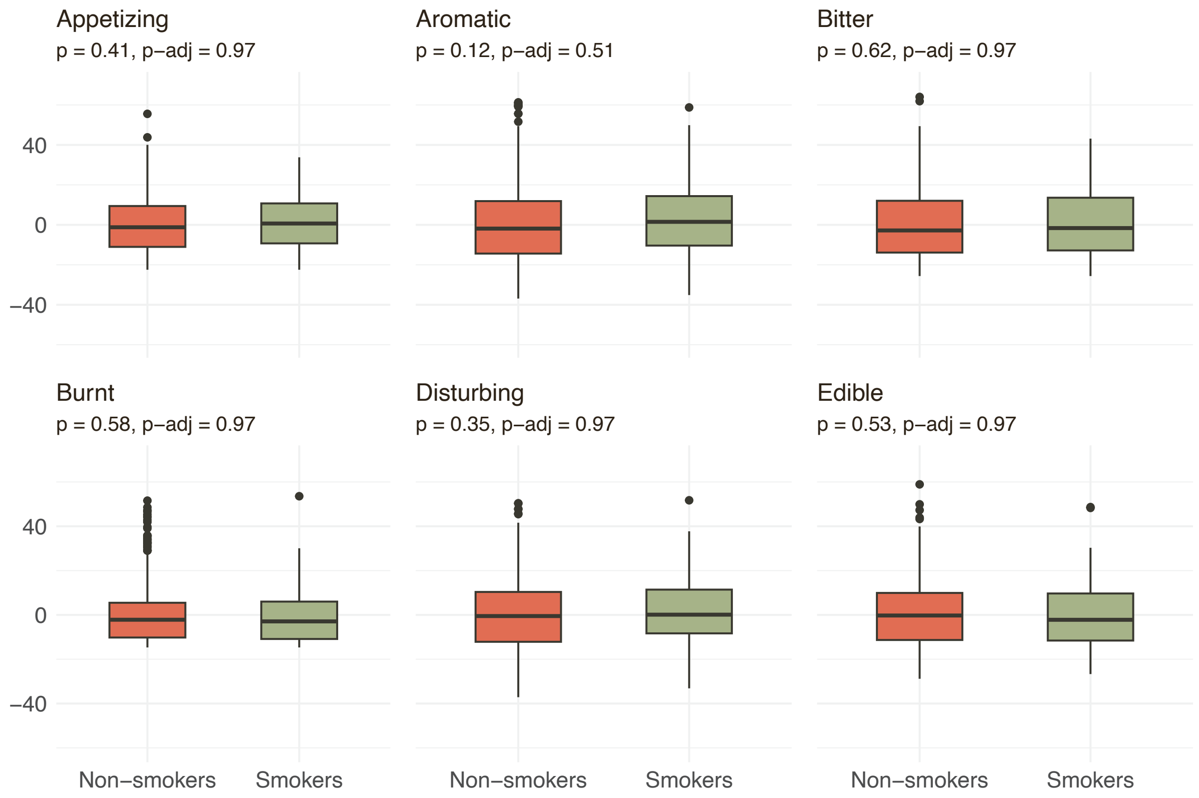

P values were adjusted using a Benjamini & Hochberg adjustment method for multiple testing.

Figure S9. Olfactory Perceptual Fingerprint descriptors feminine, intense, medicinal, mouth odor, natural, and pleasant among non-smokers (N = 796) and smokers (N = 111), related to the "Age and gender-related differences in the individual OPF descriptors" section of the results.

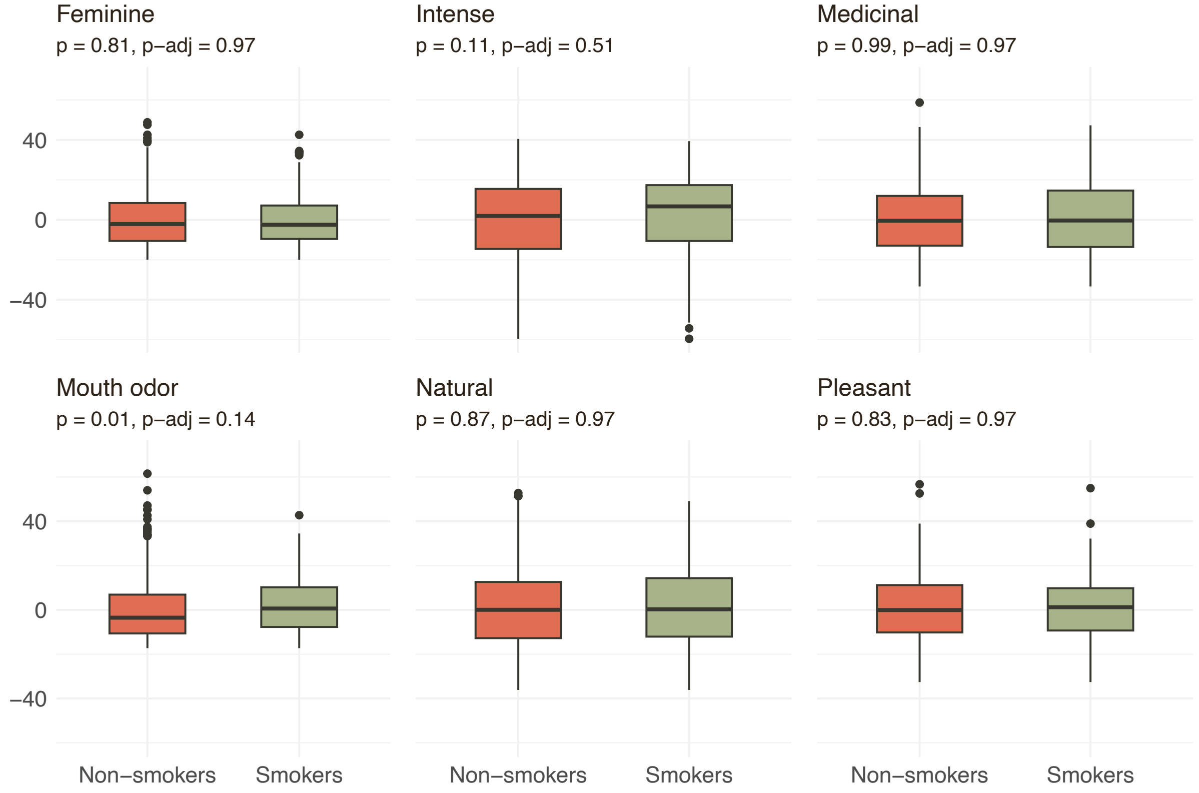

P values were adjusted using a Benjamini & Hochberg adjustment method for multiple testing.

Figure S10. Olfactory Perceptual Fingerprint descriptors appetizing and aromatic among the people younger than 30 years old (N = 519) from the 16 regions, related to the "Results remained similar among people younger than 30 years old" section of the results.

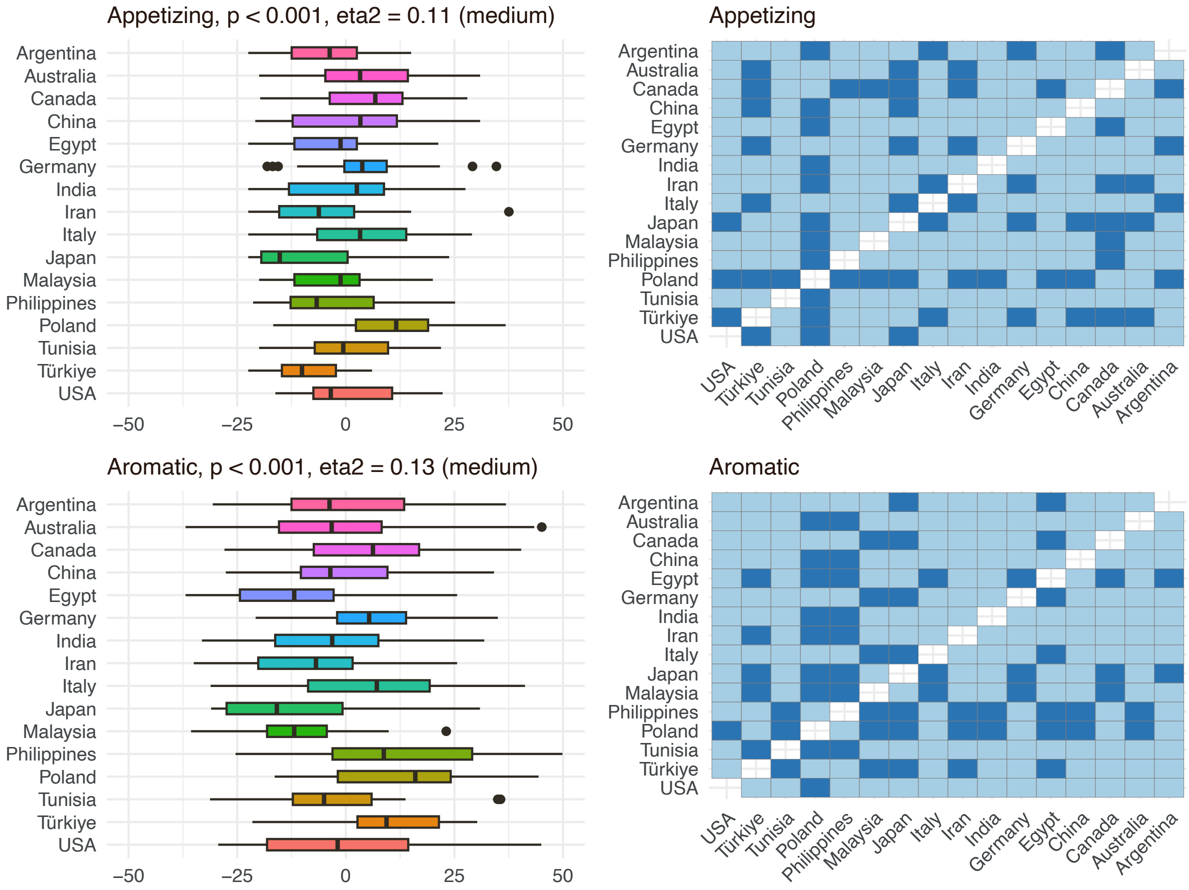

Effect size was evaluated using eta squared (eta2). On the right, post hoc analysis (Dunn test with Benjamini Hochberg adjustment for multiple testing) between the regions is shown. Dark and bright blue indicate significant and non-significant differences between the two regions, respectively.

Figure S11. Olfactory Perceptual Fingerprint descriptors bitter and burnt among the people younger than 30 years old (N = 519) from the 16 regions, related to the “Results remained similar among people younger than 30 years old” section of the results.

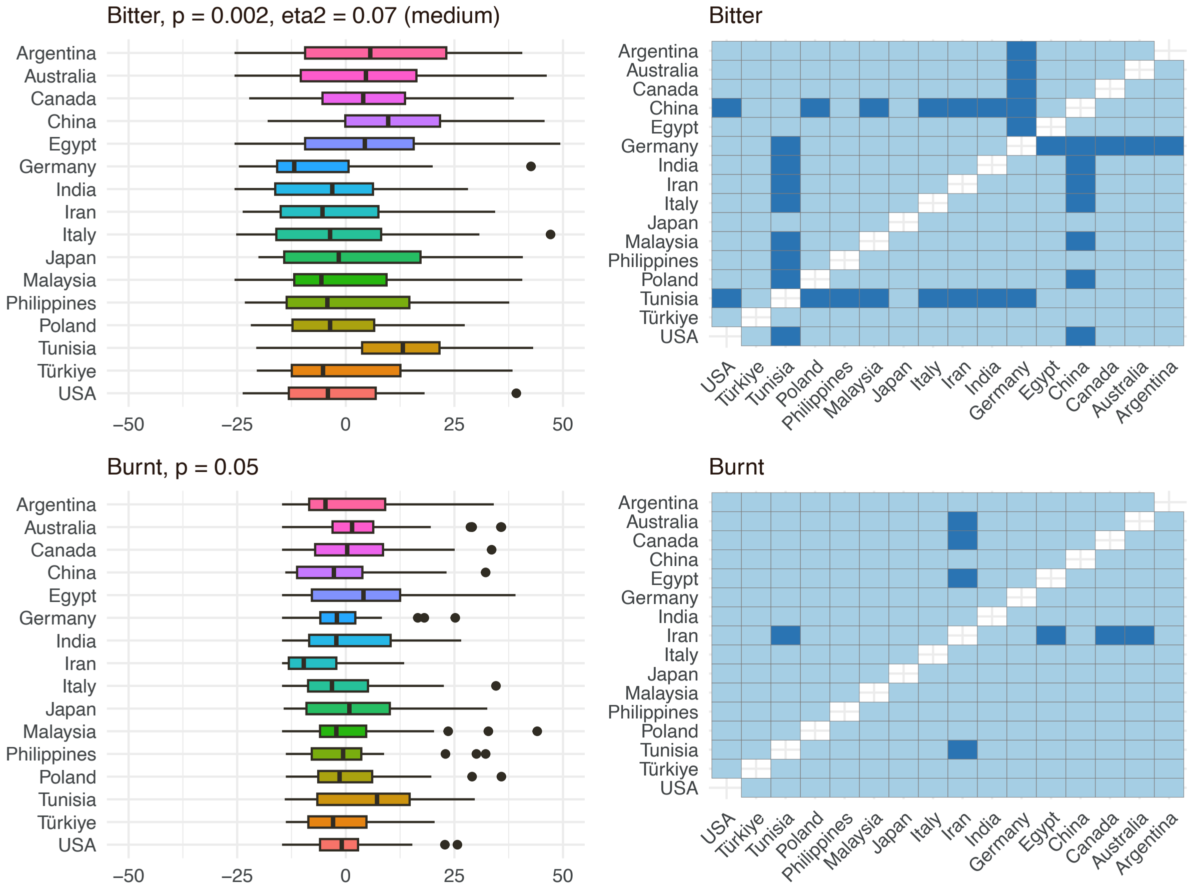

Effect size was evaluated using eta squared (eta2). On the right, post hoc analysis (Dunn test with Benjamini Hochberg adjustment for multiple testing) between the regions is shown. Dark and bright blue indicate significant and non-significant differences between the two regions, respectively.

Figure S12. Olfactory Perceptual Fingerprint descriptors disturbing and edible among the people younger than 30 years old (N = 519) from the 16 regions, related to the "Results remained similar among people younger than 30 years old" section of the results.

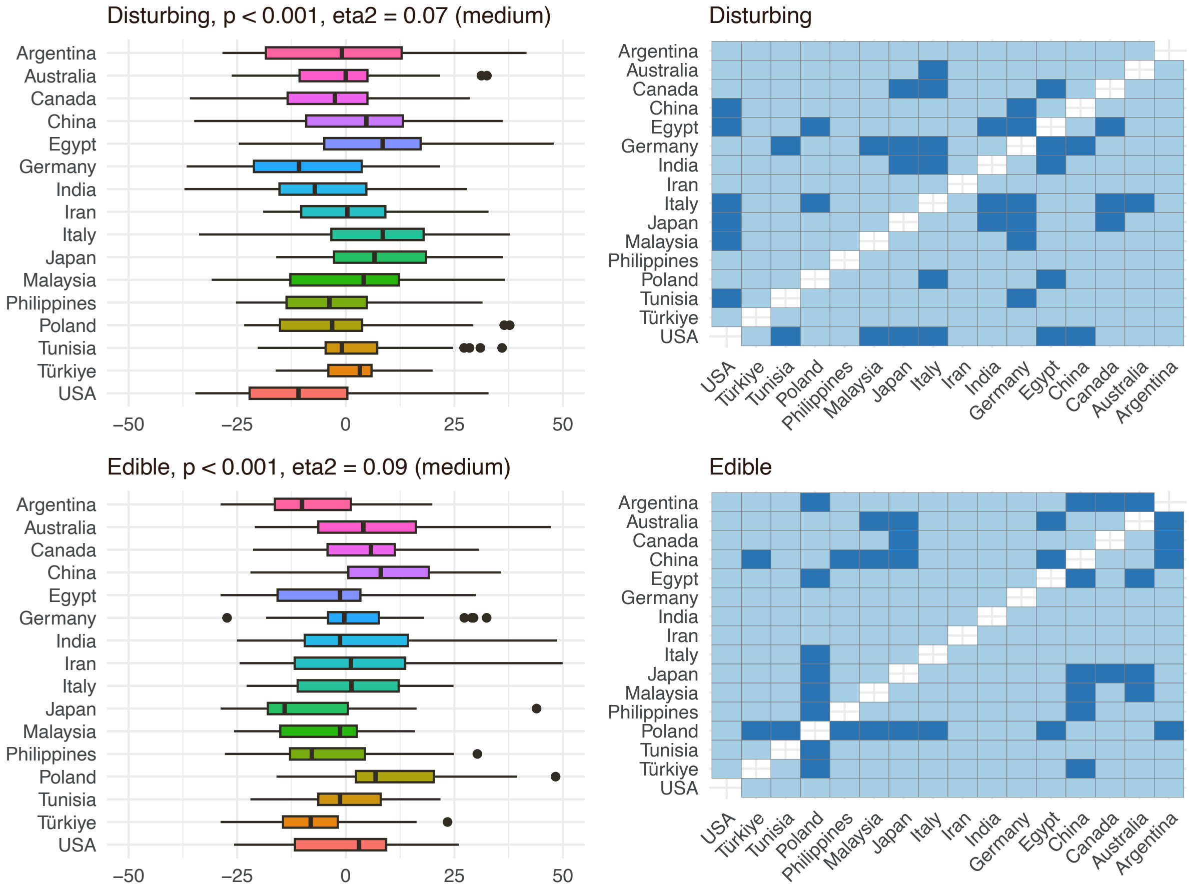

Effect size was evaluated using eta squared (eta2). On the right, post hoc analysis (Dunn test with Benjamini Hochberg adjustment for multiple testing) between the regions is shown. Dark and bright blue indicate significant and non-significant differences between the two regions, respectively.

Figure S13. Olfactory Perceptual Fingerprint descriptors feminine and intense among the people younger than 30 years old (N = 519) from the 16 regions, related to the “Results remained similar among people younger than 30 years old” section of the results.

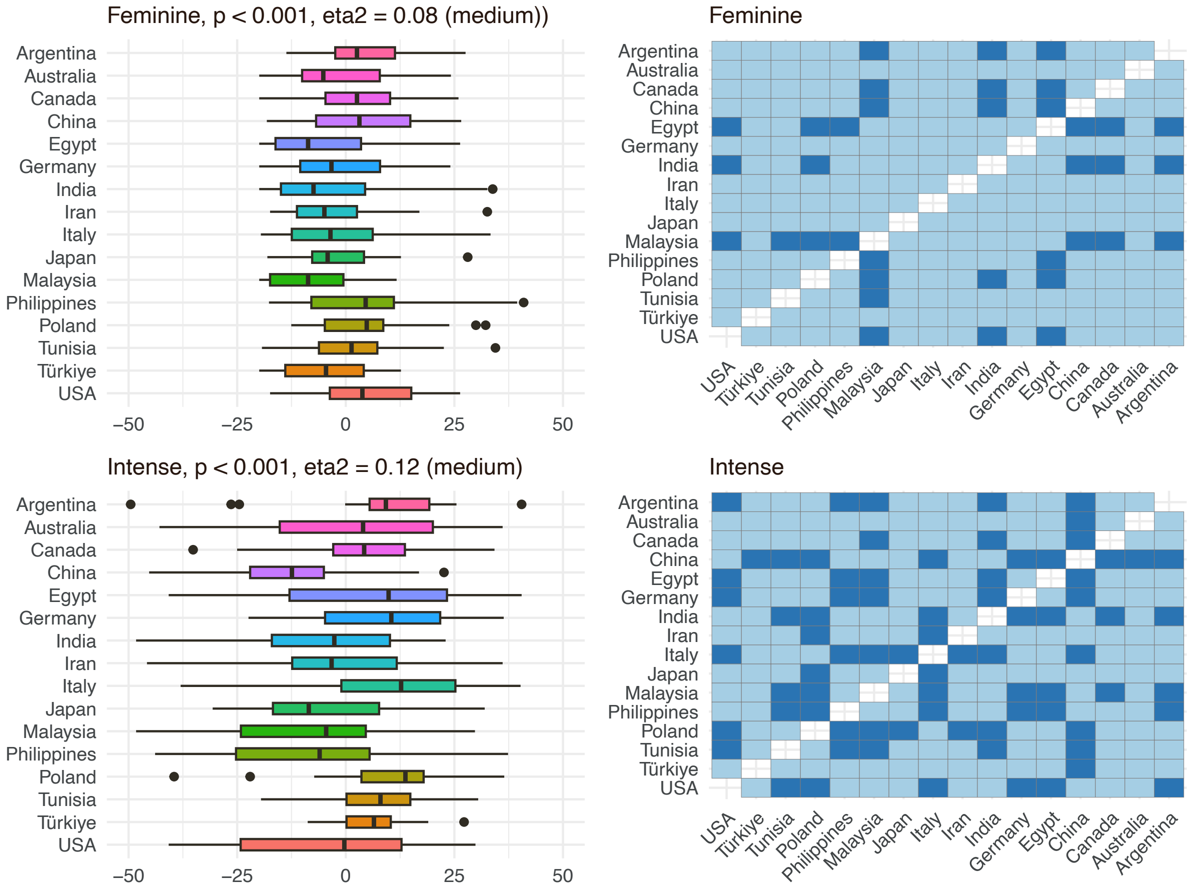

Effect size was evaluated using eta squared (eta2). On the right, post hoc analysis (Dunn test with Benjamini Hochberg adjustment for multiple testing) between the regions is shown. Dark and bright blue indicate significant and non-significant differences between the two regions, respectively.

Figure S14. Olfactory Perceptual Fingerprint descriptors medicinal and mouth-odor like among the people younger than 30 years old (N = 519) from the 16 regions, related to the "Results remained similar among people younger than 30 years old" section of the results.

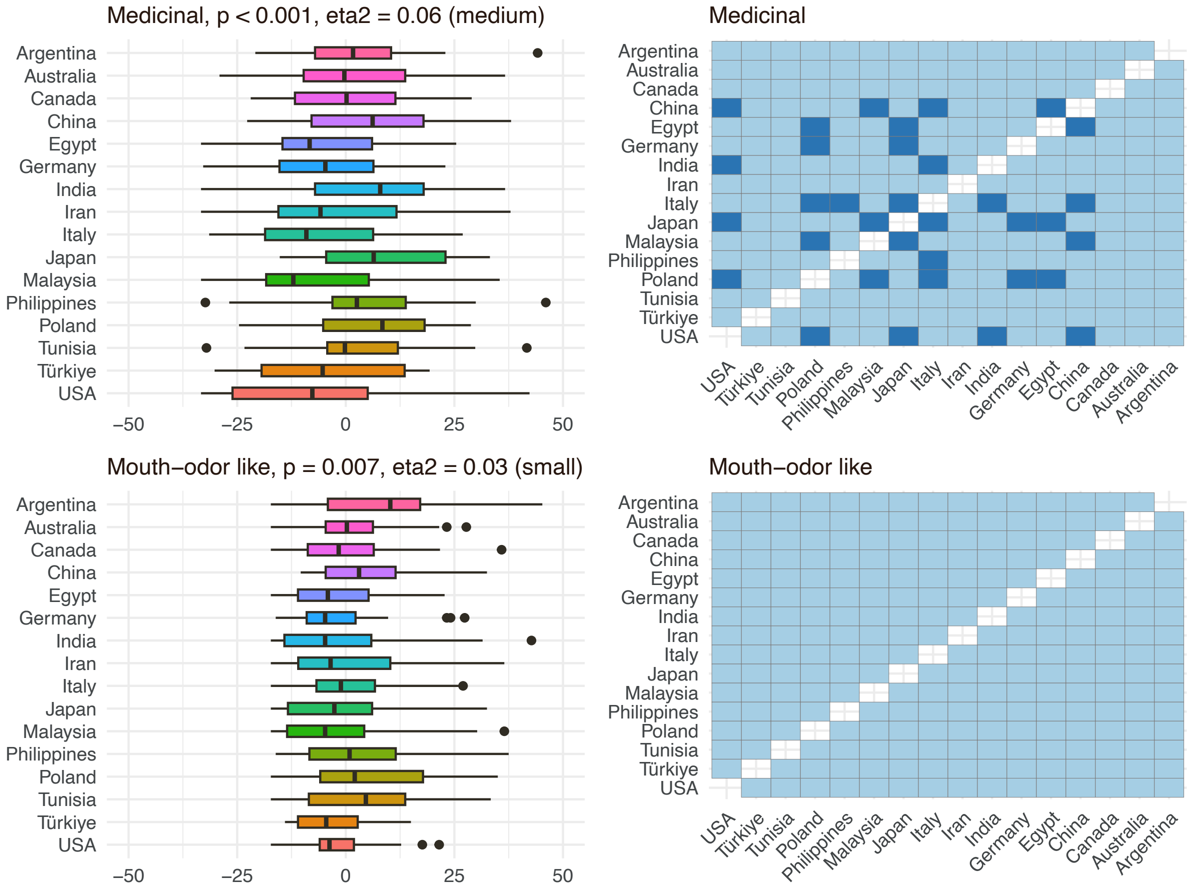

Effect size was evaluated using eta squared (eta2). On the right, post hoc analysis (Dunn test with Benjamini Hochberg adjustment for multiple testing) between the regions is shown. Dark and bright blue indicate significant and non-significant differences between the two regions, respectively.

Figure S15. Olfactory Perceptual Fingerprint descriptors natural and pleasant among the people younger than 30 years old (N = 519) from the 16 regions, related to the “Results remained similar among people younger than 30 years old” section of the results.

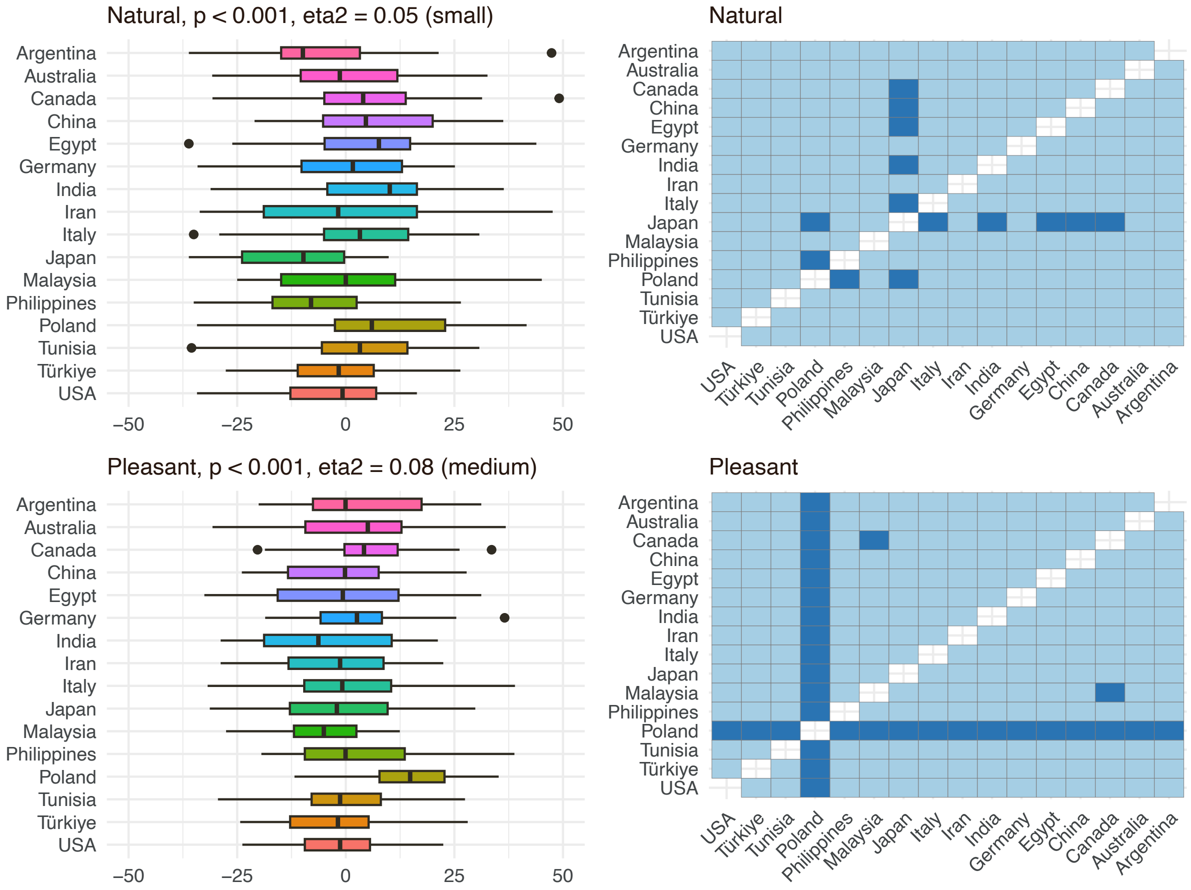

Effect size was evaluated using eta squared (eta2). On the right, post hoc analysis (Dunn test with Benjamini Hochberg adjustment for multiple testing) between the regions is shown. Dark and bright blue indicate significant and non-significant differences between the two regions, respectively.

Figure S16. Number of significant differences in the Olfactory Perceptual Fingerprint (OPF) descriptors (appetizing, aromatic etc.) between the regions among people younger than 30 years old (N = 519), related to the “Results remained similar among people younger than 30 years old” section of the results.

	USA	Türkiye	Tunisia	Poland	Philippines	Malaysia	Japan	Italy	Iran	India	Germany	Egypt	China	Canada	Australia	Argentina
Argentina	1	0	0	3	1	2	1	1	0	2	2	2	2	2	1	
Australia	0	1	0	2	1	1	2	1	2	0	1	1	1	0		1
Canada	0	1	0	1	1	5	5	1	2	2	1	4	1		0	2
China	3	3	1	5	2	4	3	3	1	2	3	4		1	1	2
Egypt	3	1	0	7	3	1	2	1	1	2	3		4	4	1	2
Germany	1	1	2	2	1	3	4	1	1	1		3	3	1	1	2
India	2	0	2	5	1	0	2	3	0		1	2	2	2	0	2
Iran	0	1	2	4	1	0	0	2		0	1	1	1	2	2	0
Italy	2	1	1	4	2	2	5		2	3	1	1	3	1	1	1
Japan	3	1	0	6	1	1		5	0	2	4	2	3	5	2	1
Malaysia	2	1	3	7	2		1	2	0	0	3	1	4	5	1	2
Philippines	0	0	3	5		2	1	2	1	1	1	3	2	1	1	1
Poland	5	3	5		5	7	6	4	4	5	2	7	5	1	2	3
Tunisia	3	1		5	3	3	0	1	2	2	2	0	1	0	0	0
Türkiye	1		1	3	0	1	1	1	1	0	1	1	3	1	1	0
USA		1	3	5	0	2	3	2	0	2	1	3	3	0	0	1

Ranging from 0, which indicates that there were no significant differences in the OPF descriptors among the two regions, up to 12, which indicates that the two regions differed in all 12 OPF descriptors.

Figure S17. Boxplots showing the distances to the centroid by region calculated using the PERMDISP2 procedure for the analysis of multivariate homogeneity of group dispersions (variances) for the 16 regions when using the Odor-Specific Olfactory Perceptual Fingerprints[S1], related to the “Results remained similar using the Odor-Specific OPF” section of the results.

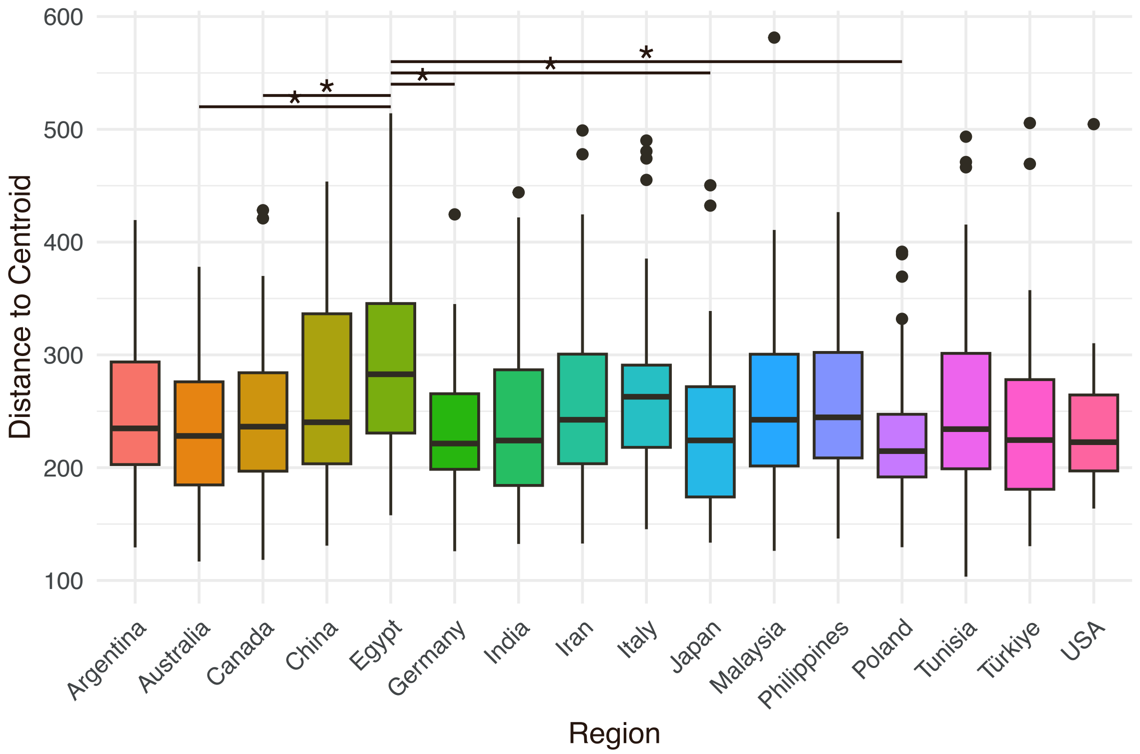

Figure S18. Results from the principal component (PC) analysis (PCA) on the Odor-Specific Olfactory Perceptual Fingerprints[S1], related to the “Results remained similar using the Odor-Specific OPF” section of the results.

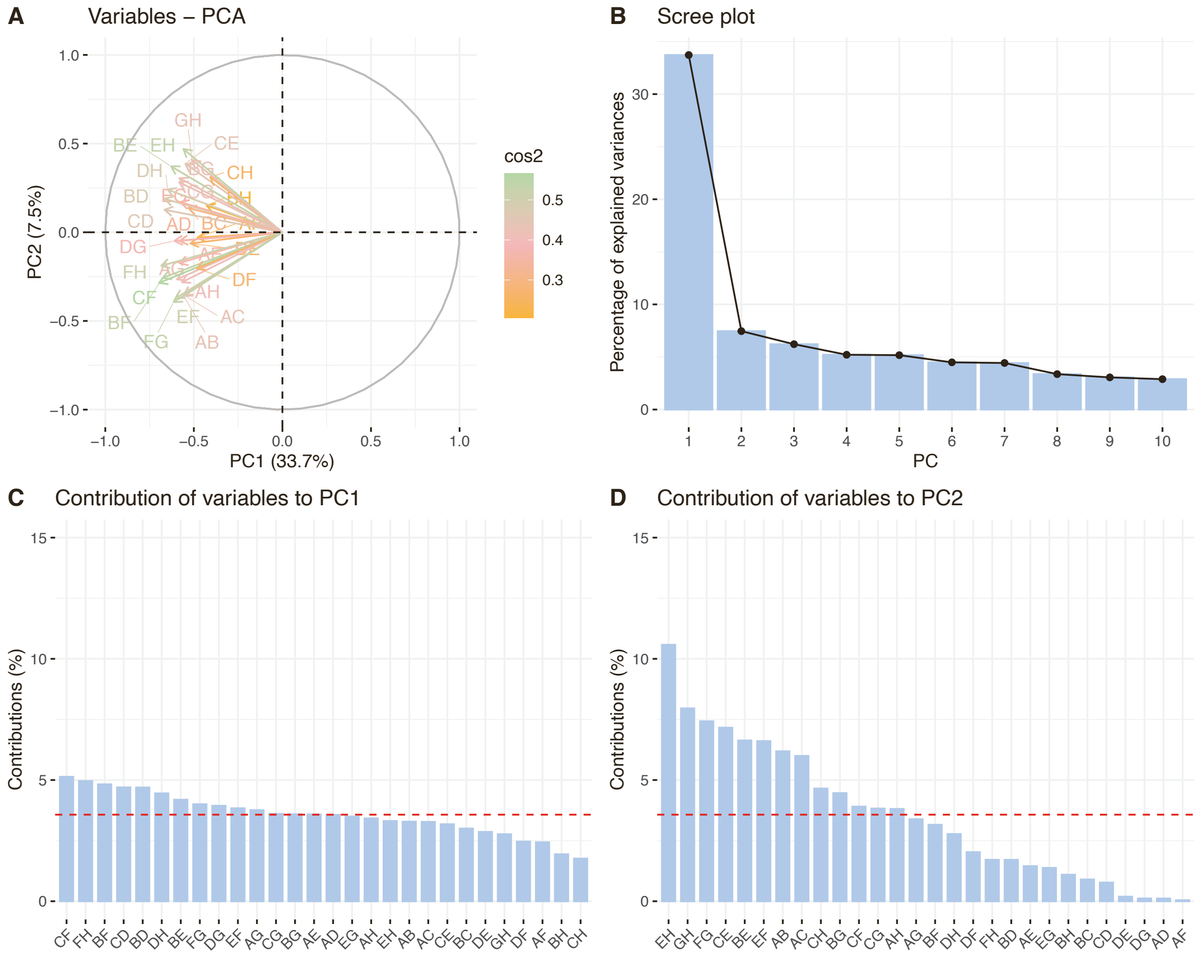

(A) Plot of the Eigenvectors of variables in PC1 and PC2.
(B) A scree plot or a bar graph of the explained variance by each PC.
(C and D) Bar graph of the contributions of each pairwise odor distance to PC1 and PC2. The red dotted line is the expected average contribution. Odors are marked from A to H as shown in Table 2.

Figure S19. Results from the principal component (PC) analysis (PCA) on the Odor-Specific Olfactory Perceptual Fingerprints[S1], related to the “Results remained similar using the Odor-Specific OPF” section of the results.

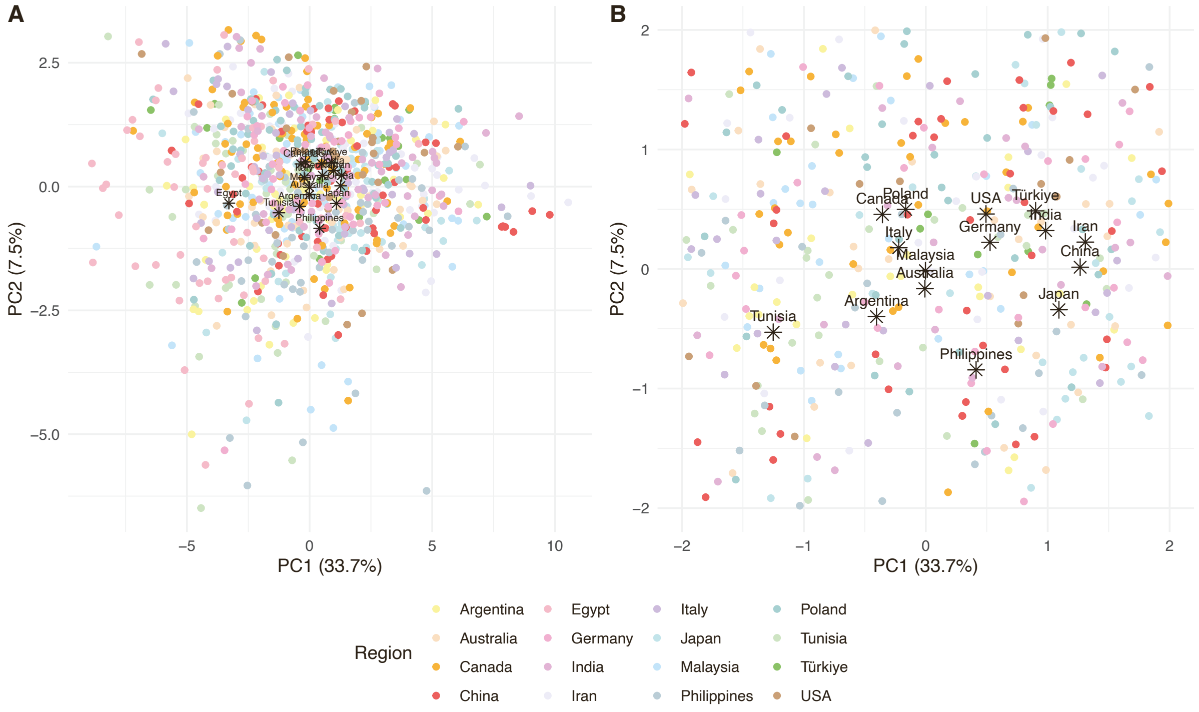

Scatterplots of the individual Odor-Specific Olfactory Perceptual Fingerprints (OPFs) plotted on a 2D graph (x axis: PC1, y axis: PC2).
On the left (A), all individuals are included. On the right (B), the centroids are additionally visualised. Of note, Egypt is out of this frame.

Table S1. Permutational multivariate analyses of variance (PERMANOVA) results with the degrees of freedom (Df), sum of squares, partial R2, pseudo-F statistic, and p value. Dependent variables were the Descriptor-Specific Olfactory Perceptual Fingerprints (12 components: appetizing, aromatic, bitter, burnt, disturbing, edible, feminine, intense, medicinal, mouth-odor like, natural, pleasant), related to the "Region-related differences explained 10% of variance in the Descriptor-Specific OPFs" section of the results.

Independent variables	Df	Sum of squares	R2	F	p value
**Region** (16 levels)	15	293038	0.100	6.6	**0.001**
**Age** (continuous value)	1	23370	0.008	7.9	**0.001**
**Gender** (2 levels)	1	9450	0.003	3.2	**0.012**
**Residual**	886	2617948	0.889		
**Total**	903[a]	2943805	1.000		

Independent variables were region (16 levels: Argentina, Australia, Canada, China, Egypt, Germany, India, Iran, Italy, Japan, Malaysia, Philippines, Poland, Tunisia, Türkiye, USA), age (continuous value), and gender (2 levels: women, men). Significance was assessed for each term sequentially from first to last.

[a]Among people without missing values for gender.

Table S2. PERMDISP2 procedure for the analysis of multivariate homogeneity of group dispersions (variances) for region and gender, when using the Descriptor-Specific Olfactory Perceptual Fingerprints, related to the "Region-related differences explained 10% of variance in the Descriptor-Specific OPFs" section of the results.

REGION					
**Independent variables**	**Df**	**Sum of squares**	**R2**	**F**	**p value**
**Region** (16 levels)	15	13391	0.05	3.2	**0.001**
**Residuals**	888	248281			
**Total**	903	261672			
GENDER					
**Independent variables**	**Df**	**Sum of squares**	**R2**	**F**	**p value**
**Gender** (2 levels)	1	0	0	0.0013	0.965
**Residuals**	902	254363			
**Total**	903	254363			

Table S3. Age and gender per region among the people younger than 30 years old, related to the "Results remained similar among people younger than 30 years old" section of the results.

**Region**	**Final sample** N (%)	**People below 30 years old** N (%)	**Age**[a] Median (25% -75%)	**Gender**[a] (women : men) N (%)
**All**	909	519	23.0 (21.0 – 26.0)	321 (62%) : 194 (37%)
Argentina (Buenos Aires)	56 (6%)	21 (4%)	27.0 (24.0 – 28.0)	8 (44%) : 10 (56%) [3]
Australia (Sydney)	59 (6%)	45 (9%)	19.0 (19.0 – 20.0)	29 (64%) : 16 (36%)
Brazil (Londrina)[b]	/			
Canada (Victoria, Trois-Rivieres)	85 (9%)	57 (11%)	22.0 (20.0-24.0)	46 (81%) : 11 (19%)
China (Beijing)	60 (7%)	32 (6%)	21.0 (20.0-22.0)	25 (78%) : 7 (22%)
Cuba (Havana)[c]	/			
Egypt (Cairo)	63 (7%)	34 (7%)	24.5 (21.3 – 28.0)	17 (50%) : 17 (50%)
Germany (Dresden)	51 (6%)	39 (8%)	24.0 (21.5 – 26.5)	21 (55%) : 17 (45%) [1]
India (Pune)	60 (7%)	35 (7%)	24.0 (21.0 – 26.0)	17 (49%) : 18 (51%)
Iran (Teheran)	58 (6%)	23 (4%)	23.0 (21.0 – 25.5)	17 (74%) : 6 (26%)
Italy (Cagliari)	58 (6%)	45 (9%)	24.0 (22.0 – 26.0)	19 (42%) : 26 (58%)
Japan (Tokyo)	53 (6%)	19 (4%)	26.0 (24.0 – 27.0)	10 (53%) : 9 (47%)
Malaysia (Kuala Lumpur)	64 (7%)	29 (6%)	24.0 (23.0 – 28.0)	21 (72%) : 8 (28%)
Philippines (Manila)	59 (6%)	29 (6%)	27.0 (25.0 – 28.0)	15 (52%) : 14 (48%)
Poland (Wroclaw)	65 (7%)	40 (8%)	22.0 (21.0 – 25.0)	27 (68%) : 13 (32%)
Tunisia (Tunis)	60 (7%)	31 (6%)	26.0 (23.0 – 28.0)	22 (71%) : 9 (29%)
Türkiye (Izmir)	27 (3%)	15 (3%)	20.0 (19.0 – 21.5)	9 (60%) : 6 (40%)
USA (Chicago)	31 (3%)	25 (5%)	23.0 (21.0 – 24.0)	18 (72%) : 7 (28%)
**P value (effect size)**			**< 0.001 (eta2 = 0.2)**	**0.003**

[a]Among people younger than 30 years old.
[b]Omitted due to the missing odor pleasantness values.
[c]Omitted due to the small sample size.
[] – number of missing values,
CROCUS - CROss-CUltural Study on Variability in Chemosensory Sensitivity

Table S4. Permutational multivariate analyses of variance (PERMANOVA) results with the degrees of freedom (Df), sum of squares, partial R2, pseudo-F statistic, and p value including only people younger than 30 years old (N = 514). Dependent variables were the Descriptor-Specific Olfactory Perceptual Fingerprints (12 components: appetizing, aromatic, bitter, burnt, disturbing, edible, feminine, intense, medicinal, mouth-odor like, natural, pleasant), related to the “Results remained similar among people younger than 30 years old” section of the results.

Independent variables	Df	Sum of squares	R2	F	p value
**Region** (16 levels)	15	167792	0.108	4.1	**0.001**
**Gender** (2 levels)	1	11378	0.007	4.1	**0.003**
**Residual**	498	1367825	0.884		
**Total**	514[a]	1546996	1.000		

Independent variables were region (16 levels: Argentina, Australia, Canada, China, Egypt, Germany, India, Iran, Italy, Japan, Malaysia, Philippines, Poland, Tunisia, Türkiye, USA), and gender (2 levels: women, men). Significance was assessed for each term sequentially from first to last.
[a]Among people without missing values for gender.

Table S5. Permutational multivariate analyses of variance (PERMANOVA) results with the degrees of freedom (Df), sum of squares, partial R2, pseudo-F statistic, and p value. Dependent variables were the Odor-Specific Olfactory Perceptual Fingerprints[S1] (28 components or pairwise odor distances, see Figure S1), related to the "Results remained similar using the Odor-Specific OPF" section of the results.

Independent variables	Df	Sum of squares	R2	F	p value
**Age** (continuous value)	1	302597	0.005	4.4	**0.003**
**Gender** (2 levels)	1	180076	0.003	2.6	**0.013**
**Region** (16 levels)	15	5489095	0.082	5.3	**0.001**
**Residual**	886	60809575	0.911		
**Total**	903[a]	66781344	1.000		

Independent variables were age (continuous), gender (2 levels: women, men), and region (16 levels: Argentina, Australia, Canada, China, Egypt, Germany, India, Iran, Italy, Japan, Malaysia, Philippines, Poland, Tunisia, Türkiye, USA). Significance for each independent variable was evaluated sequentially from first to last.

[a]Among people without missing values for gender.

Table S6. PERMDISP2 procedure for the analysis of multivariate homogeneity of group dispersions (variances) for region and gender when using the Odor-Specific Olfactory Perceptual Fingerprints[S1], related to the "Results remained similar using the Odor-Specific OPF" section of the results.

REGION					
**Independent variables**	**Df**	**Sum of squares**	**R2**	**F**	**p value**
**Region** (16 levels)	15	222688	0.04	2.8	**0.002**
**Residuals**	888	4802395			
**Total**	903				
GENDER					
**Independent variables**	**Df**	**Sum of squares**	**R2**	**F**	**p value**
**Gender** (2 levels)	1	394	-	0.06	0.82
**Residuals**	902	5531142			
**Total**	903				

Table S7. References for the effect sizes, relates to Figures 4a – 4f, Figure 5, Figures S7a – S7f, Figure S5a and S5b.

	**Small effect size**	**Medium effect size**	**Large effect size**
Eta-squred[S2] (effect size for Kruskal Wallis analysis of variance)[a]	0.01 to 0.06	0.06 to 0.14	> 0.14
Vargha and Delaney's A[S3] (effect size for Mann-Whitney test)[b]	0.56 to 0.64 and 0.34 to 0.44	0.64 to 0.71 and 0.29 to 0.34	> 0.71 and < 0.29
ρ values[S4] (Spearman correlation coefficients)[c]	0.1 to 0.3	0.3 to 0.5	> 0.5

[a] Figures 4a – 4f, Figure 5, Figures S7a – S7f.
[b] Figure S5a, S5b.
[c] Results section.

Supplemental references

S1. Levin, N., Frumin, I., Bar-Zvi, D., Shushan, S., and Sobel, N. (2015). Individual olfactory perception reveals meaningful nonolfactory genetic information. Proc Natl Acad Sci U S A 112, 8750-8755. 10.1073/pnas.1424826112.

S2. Kassambara, A. (2023). rstatix: Pipe-Friendly Framework for Basic Statistical Tests. https://rpkgs.datanovia.com/rstatix/.

S3. Torchiano, M. (2020). effsize: Efficient Effect Size Computation. https://CRAN.R-project.org/package=effsize.

S4. Cohen, J. (1988). Statistical power analysis for the behavioral sciences, 2nd Edition (L. Erlbaum Associates).
